# Carbon Fixation in the Chemolithoautotrophic Bacterium *Aquifex aeolicus* Involves Two Low-Potential Ferredoxins as Partners of the PFOR and OGOR Enzymes

**DOI:** 10.3390/life13030627

**Published:** 2023-02-23

**Authors:** Laura Prioretti, Giulia D’Ermo, Pascale Infossi, Arlette Kpebe, Régine Lebrun, Marielle Bauzan, Elisabeth Lojou, Bruno Guigliarelli, Marie-Thérèse Giudici-Orticoni, Marianne Guiral

**Affiliations:** 1 CNRS, Bioénergétique et Ingénierie des Protéines, Aix Marseille Université, IMM, 13009 Marseille, France; 2 CNRS, Aix Marseille Université, IMM, 13009 Marseille, France

**Keywords:** carbon fixation, reverse TCA cycle, low potential ferredoxin, pyruvate:ferredoxin oxidoreductase, oxoglutarate:ferredoxin oxidoreductase, Wood-Ljungdahl pathway, chemolithoautotrophic bacteria, hyperthermophilic bacteria, hydrogenase

## Abstract

*Aquifex aeolicus* is a microaerophilic hydrogen- and sulfur -oxidizing bacterium that assimilates CO_2_ via the reverse tricarboxylic acid cycle (rTCA). Key enzymes of this pathway are pyruvate:ferredoxin oxidoreductase (PFOR) and 2-oxoglutarate:ferredoxin oxidoreductase (OGOR), which are responsible, respectively, for the reductive carboxylation of acetyl-CoA to pyruvate and of succinyl-CoA to 2-oxoglutarate, two energetically unfavorable reactions that require a strong reduction potential. We have confirmed, by biochemistry and proteomics, that *A. aeolicus* possesses a pentameric version of these enzyme complexes ((αβγδε)_2_) and that they are highly abundant in the cell. In addition, we have purified and characterized, from the soluble fraction of *A. aeolicus*, two low redox potential and oxygen-stable [4Fe-4S] ferredoxins (Fd6 and Fd7, E^0^ = −440 and −460 mV, respectively) and shown that they can physically interact and exchange electrons with both PFOR and OGOR, suggesting that they could be the physiological electron donors of the system in vivo. Shotgun proteomics indicated that all the enzymes assumed to be involved in the rTCA cycle are produced in the *A. aeolicus* cells. A number of additional enzymes, previously suggested to be part of a putative partial Wood-Ljungdahl pathway used for the synthesis of serine and glycine from CO_2_ were identified by mass spectrometry, but their abundance in the cell seems to be much lower than that of the rTCA cycle. Their possible involvement in carbon assimilation is discussed.

## 1. Introduction

Early microorganisms evolved different types of CO_2_ fixation pathways in order to cope with all the forms of energy naturally available on Earth and possibly to ensure energy conservation and recycling. This is the case of the reverse (or reductive) tricarboxylic acid cycle (rTCA cycle) [1,2,3]. In the rTCA cycle, two molecules of CO_2_ are fixed through the reductive carboxylation of acetyl-CoA to pyruvate and of succinyl-CoA to 2-oxoglutarate. These reactions are catalyzed by two ferredoxin (Fd)-dependent signature enzymes: the pyruvate:Fd oxidoreductase (PFOR) and the 2-oxoglutarate:Fd oxidoreductase (OGOR), respectively [2,3]. Both enzymes belong to the thiamine pyrophosphate (TPP)-dependent 2-oxoacid:Fd oxidoreductase (OFOR) superfamily [3,4] and perform reversible reactions. The one catalyzed by PFOR has been thoroughly characterized in the direction of the CoA thioester biosynthesis in a variety of extremophile microorganisms. The only rTCA user in which PFOR and OGOR reactions were characterized is *Hydrogenobacter thermophilus*, a deep-branching member of the Aquificaceae family [5,6,7,8,9,10]. The challenge resides in the fact that the reductive carboxylation reactions require a strong reducing power that can be provided by low potential Fds. In *H. thermophilus*, two [4Fe-4S] Fds, Ht-Fd1 (E^0^ = −485 mV) and Ht-Fd2, have been characterized [11,12] and shown to be the physiological electron donors of the pathway [6,8]. *Aquifex aeolicus*, a close relative of *H. thermophilus*, is an obligate chemolithoautotroph performing CO_2_ fixation via the rTCA cycle. It possesses genes coding for the five-subunit PFOR and OGOR and for up to seven Fds [13,14]. Four of these (Fd2, Fd3, Fd6, and Fd7) contain [4Fe-4S] clusters [11,13] and are thus supposed to have a low redox potential, which is required for assuring the rTCA cycle. In addition, based on the in silico reconstruction of *A. aeolicus* metabolic pathways, it was proposed that it may use a unique and previously unrecognized hybrid form of carbon fixation with the use, in parallel to the rTCA cycle, of a pathway composed of a partial form of the reductive acetyl-CoA pathway (more precisely, an incomplete Wood-Ljungdahl pathway that was called “direct one-carbon group reduction”) in association with the reversible glycine cleavage system to synthesize a small set of molecules, including glycine and serine [1,15,16]. On the other hand, the glycine cleavage system in *H. thermophilus* does not appear to be operational [17]. This demonstrates that carbon metabolism in the Aquificales is only partially understood, leaving room for new and potentially transposable discoveries.

In this manuscript, we attempt to demonstrate the functionality of the rTCA cycle in *A. aeolicus* by showing the characteristics of its principal components (PFOR, OGOR, and Fds) and the interactions between them, as well as the presence of all other enzymes putatively involved in this pathway. We provide hints on the possible existence of an incomplete Wood-Ljungdahl pathway working in parallel to the rTCA cycle and alternative serine and glycine biosynthesis routes.

## 2. Materials and Methods

### 2.1. A. aeolicus Growth Condition

*A. aeolicus* VF5 cells were routinely grown, as previously described, in the presence of hydrogen (80 mmol/flask) and thiosulfate (S_2_O_3_^2−^, 1 g L^−1^) and harvested in the late exponential phase [18].

### 2.2. Enrichment of PFOR and OGOR from A. aeolicus Soluble Fraction

All buffers used during protein extraction and fractionation steps were continuously bubbled under argon and contained 1 mM dithiotreitol (DTT) and 1 mM sodium dithionite (DTH) to remove any oxygen traces. *A. aeolicus* cells (50 g wet weight) were resuspended in 50 mM Tris-HCl at pH 7.6, a protease inhibitor cocktail (SIGMAFAST tablets), and 5% (*v*/*v*) glycerol, then disrupted with a French press. After exclusion of cell debris by gentle centrifugation at 5000× *g* for 10 min, total proteins were ultracentrifuged at 100,000× *g* for 1 h.

Soluble proteins were separated by two affinity chromatography steps using a FPLC apparatus (Amersham Pharmacia Biotech, UK): (1) The supernatant was loaded onto a DEAE (Diethylaminoethyl)-cellulose column, equilibrated with 50 mM Tris-HCl at pH 7.6, and eluted with a stepwise gradient of increasing concentrations of NaCl. Ten fractions with A_280_ ≥ 0.6 were obtained: one of them, eluted at 150 mM NaCl, contained most of the PFOR activity; a second fraction, eluted at 300 mM NaCl, contained most of the OGOR activity and was loaded onto a second column; (2) the fraction was loaded onto a Bio-Gel-HTP column equilibrated with 50 mM Tris-HCl at pH 7.6 and 300 mM NaCl to obtain an enriched fraction of OGOR activity. The proteins were eluted with a stepwise gradient of increasing concentrations of phosphate buffer at pH 7. Four fractions with A_280_ ≥ 0.4 were obtained; two of them, eluted at phosphate buffer concentrations of 340 and 375 mM, respectively, contained most of the OGOR activity. All fractions were stored anaerobically at −80 °C after addition of 10% glycerol, 1 mM DTT, and 1 mM DTH.

### 2.3. Purification of Native Ferredoxins

50 g of *A. aeolicus* cells were harvested in the late exponential phase, then resuspended in 50 mM Tris-HCl at pH 7.6, 5 mM EDTA, 0.5 mM PMSF, and 10 µg/mL Deoxyribonuclease I and disrupted with a French press. The native Fds (Ferredoxin 6 (Fd6) and Ferredoxin 7 (Fd7)) were purified aerobically from the soluble fraction following ultracentrifugation at 100,000× *g* for 1 h, and their purity was verified by gel electrophoresis. Proteins were separated in four chromatography steps using a FPLC apparatus (Amersham Pharmacia Biotech, UK). All soluble proteins were loaded onto a DEAE-cellulose column, equilibrated with 50 mM Tris-HCl at pH 7.6, and eluted with a stepwise gradient of increasing concentrations of NaCl. Both Fds were eluted together at 300 mM NaCl and were subsequently loaded onto a Bio-Gel–HTP column equilibrated with 50 mM Tris-HCl at pH 7.6 and 300 mM NaCl. The proteins were eluted with a stepwise gradient of increasing concentrations of phosphate buffer at pH 7. Both Fds were eluted together once more in 50 mM phosphate buffer before being separated by gel filtration in a Superose 12 column equilibrated with 50 mM Tris-HCl at pH 7.6 and 100 mM NaCl. After dialysis, the fractions containing both Fds were pooled together and loaded onto a Mono Q column equilibrated with 50 mM Tris-HCl at pH 7.6. Proteins were eluted with a linear gradient of NaCl. Two distinct fractions containing Fd6 and Fd7 were eventually eluted, respectively, at 500 and 540 mM NaCl, concentrated in Vivaspin centrifugal concentrators (molecular mass cutoff of 3000 Da), and stored at −80 °C in the presence of 10% glycerol.

### 2.4. Heterologous Expression and Purification of Recombinant Fd7

*A. aeolicus* Fd7 gene was cloned, and the protein overexpressed and purified as described by Kpebe et al. [19]. Briefly, the Fd7 gene (*fdx*7 [11]; NCBI protein ID: WP_164930743.1) was amplified using the primers Fd7-aqui-for (5′-AATTGGATCCAGGAGGTTAGTTAGAATGGCAAAATTAAAGACCATG-3′), which introduced a BamHI RE site, and Fd7-aqui-rev (5′-AATTCCCGGGCTCTTCGAGTTCTTCAACGAT-3′), which introduced a SmaI RE site. The amplified fragment was subcloned into the pGEM-T Easy vector and then inserted into the shuttle vector system described by Gauquelin et al. [19,20] upstream of the Strep-tag II sequence. The resulting plasmid was then introduced into the *E. coli* MG1655 ΔiscR::kan strain by electroporation [21]. Cells were grown anaerobically in 2 L gas-tight bottles at 37 °C in terrific broth supplemented with FeSO_4_ 0.1 g L^−1^, L-cysteine 0.1 g L^−1^, Fe(III) citrate 0.1 g L^−1^ and ammonium Fe(III) citrate 0.1 g L^−1^; pH 7 was kept constant with phosphate buffer. After 23 h, cells were harvested and disrupted with a French press as described in [19]. After ultracentrifugation at 100,000× *g* for 1 h, the soluble fraction was loaded onto a 5 mL Strep-Tactin Superflow affinity column (IBA Life Sciences, Göttingen, Germany), and Fd7 carrying the Strep-tag was purified according to the manufacturer’s instructions. For the anaerobic purification of the Fd7Strep, the *E. coli* soluble fraction was prepared by suspending the cells in 0.1 M Tris-HCl at pH 8 and 0.15 M NaCl with protease inhibitors (SIGMAFAST tablets) and deoxyribonuclease I (1 µg mL^−1^), breaking them in a glove box using a sonicator (12 cycles of 1 min with a 1-min pause), and then centrifuging them at 45,000× *g* for 45 min. The purification on the Strep-Tactin column was performed in a glove box. Protein purity was verified by 15% SDS-PAGE; all fractions containing Fd7Strep were pooled, then dialyzed and concentrated using Vivaspin centrifugal concentrators (molecular mass cutoff of 3000 Da).

### 2.5. Protein Concentration, Sequencing, Spectroscopy, Electrochemistry, and MALDI-TOF Mass Spectrometry

Protein concentration was determined with the Bicinchoninic Acid Protein Assay Kit (Sigma Aldrich, Saint-Quentin-Fallavier, France) using Bovine Serum Albumin as a standard.

N-terminal sequence determination was performed by stepwise Edman degradation using an automatic sequencer model, Procise 494 from Applied Biosystems, as previously described [22].

Fds optical spectra, of proteins diluted in 0.15 M NaCl and 0.1 M Tris-HCl at pH 8 and, were recorded at room temperature using a Cary 60 UV-Vis Spectrophotometer.

EPR spectroscopy experiments were performed on a Bruker ELEXSYS E500 spectrometer equipped with an ER4102ST standard rectangular cavity fitted to an Oxford Instruments ESR 900 helium flow cryostat. Spin intensity measurements were performed by double integration of EPR spectra recorded under non-saturating conditions and compared to a 1 mM Cu(II)-EDTA reference sample.

Cyclic voltammetry (CV) and square-wave voltammetry (SWV) measurements with a pyrolytic graphite membrane working electrode were carried out using an EG&G 273 potentiostat modulated by EG&G PAR M 270/250 software. The CV scan rate was generally 20 mV s^–1^. SWV curves were obtained using 5 Hz as the square-wave frequency, 2 mV as the scan increment, and 25 mV as the pulse height amplitude. As previously described, direct electrochemistry of Fds was obtained by the addition of Poly(L-lysine) hydrobromide aliquots [23].

The molecular mass determination of native Fd6 and Fd7 was performed by MALDI-TOF mass spectrometry on a reflection time of flight mass spectrometer equipped with delayed extraction (Voyager DE-RP, Perseptive Biosystems Inc., Framingham, MA, USA). 0.7 µL of sample were directly mixed on the support with an equal volume of matrix (saturated solution of sinapinic acid in acetonitrile 40%, water with 0.1% of trifluoroacetic acid 60%).

### 2.6. Gel Electrophoreses

Denaturing (15% acrylamide with a 5% acrylamide stacking gel) and native (10% acrylamide with a 5% acrylamide stacking gel) Tris-Glycine polyacrylamide gel electrophoreses were performed as previously described [24]. For the denaturing gel, the samples containing SDS and β-mercaptoethanol were boiled for 5 min before being loaded onto the gel.

Blue native (BN) gels (3–12% without a stacking gel) were performed as previously described [24]. After migration, proteins were stained with Coomassie blue R-250, and PFOR and OGOR enzyme activities were detected by incubation of the gel in 10 mL of reaction buffer (50 mM glycine at pH 9, either 2-oxoglutarate or pyruvate at 10 mM, 0.1 mM coenzyme A, 0.2 mM TPP, 1 mM MgCl_2_, and 5 mM methyl viologen (MeV)). The reaction buffer was extensively degassed with argon, and the reaction was performed at 80 °C. Triphenyltretrazolium chloride at 5 mM, which becomes red when reduced by MeV, was added at the end of the reaction.

### 2.7. Enzymatic Activities

OGOR and PFOR activities (decarboxylation direction) were routinely spectrophotometrically assayed by following the MeV reduction at 604 nm (ε = 13,600 M^−1^ cm^−1^) under an argon atmosphere at 80 °C. The reaction mixture (final volume 500 µL), pre-incubated under argon bubbling at 80 °C for 5 min, contained a cocktail of buffers at pH 8 (25 mM MES, 25 mM HEPES, 25 mM Tricine, and 25 mM AMPSO), either 2-oxoglutarate or pyruvate at 10 mM, 0.1 mM coenzyme A, 0.2 mM TPP, 1 mM MgCl_2_, and 5 mM MeV. The reaction started with the addition of the protein extract (20–50 µg total proteins). The specific activity corresponds to the reduction of 1 µmol of Mev per minute and per mg.

The *A. aeolicus* soluble fraction, used to determine the optimal temperature and pH of PFOR and OGOR, was prepared by suspending the cells in 25 mM Tris-HCl at pH 7.6 with protease inhibitors (SIGMAFAST tablets), breaking them anaerobically (in a glove box), using a BeadBeater homogenizer (BioSpec, Bartlesville, OK, USA; 3 cycles of 90 s), and centrifuging them at 14,000× *g* for 5 min. For the optimum pH determination, the same cocktail of buffers was used but adjusted from pH 6 to pH 9.5 with intervals of 0.5 pH units, at 80 °C. To obtain the optimal temperature of the two enzymes, the reactions were measured from 20 to 85 °C using the previous reaction mixture, except that the concentration of 2-oxoglutarate or pyruvate was 5 mM and the buffer was 25 mM phosphate at pH 7.

To compare PFOR and OGOR activities under various extraction/assay conditions, the soluble fraction was prepared either anaerobically (in a glove box) as just described or partially aerobically using the same protocol except that the cells were suspended in a buffer extensively degassed with argon and broken on the bench. The assay (decarboxylation reaction) was performed as described above, in the presence or absence of reducing agents (1 mM DTH and 1 mM DTT).

The reaction of Fd6 and Fd7Strep with OGOR and PFOR (decarboxylation direction), considering the capacity of OGOR and PFOR to transfer electrons to the Fd, was assayed spectrophotometrically at 80 °C by following the Fd-mediated reduction of 0.1 mM metronidazole (MTZ) at 322 nm (ε = 9300 M^−1^ cm^−1^) [4]. The reaction medium is the same as the previous one, except that it is at pH 9 and does not contain MeV but MTZ. The reaction started with the addition of the enriched fraction of either OGOR (12.5–50 µg total proteins) or PFOR (50–100 µg total proteins) and of either native Fd6 or recombinant Fd7 (10 µM). Control tests were performed in the absence of Fd or coenzyme A. The specific activity corresponds to the reduction of 1 µmol of MTZ per minute and per mg. To test whether hydrogenases can reduce Fd6 and Fd7Strep, the reduction of MTZ by these two Fds was tested in the presence of either the periplasmic dimeric [NiFe] Hyn hydrogenase from *Desulfovibrio fructosovorans* [25] at 50 °C, in 10 mM MOPS buffer at pH 6.8 saturated with H_2_, or the periplasmic Hydrogenase I from *A. aeolicus* [26] at 80 °C, in 50 mM HEPES pH 7 saturated with H_2_.

For all these assays, the background chemical reactions of MeV or MTZ reduction by DTT and DTH were negligible.

### 2.8. Pull-Down Assay

To detect the interaction between PFOR or OGOR and Fd7Strep, a pull-down experiment was performed in which the purified tagged Fd7 was incubated with a soluble cell extract of *A. aeolicus* before being further purified, on a Strep-Tactin resin column. 2 g of *A. aeolicus* cells, stored at −80 °C, were re-suspended in 3 mL of 0.1 M Tris-HCl at pH 8, previously degassed with argon, containing Deoxyribonuclease I (10 µg mL^−1^) and protease inhibitors (cOmplete^TM^ ULTRA Tablets, Roche, Bâle, Suisse), and broken twice with a cell disrupter (Constant system Ltd., Northants, UK) at 1.6 kbar. After a short spin at 3200× *g* for 8 min, the crude extract was ultracentrifuged at 150,000× *g* for 45 min to obtain the total soluble extract. 500 µL to 1 mL of the soluble extract (13 to 20 µg µL^−1^) was incubated with 100 µL (450 µg) of Fd7Strep and avidin (to prevent biotinylated proteins from the soluble extract of *A. aeolicus* from binding to Strep-Tactin resin) for 1 h at room temperature, under semi-anaerobic conditions. After addition of NaCl to a final concentration of 0.15 M, the mixture was passed through a Strep-Tactin column (500 µL, Superflow High Capacity resin, IBA Lifesciences GmbH, Göttingen, Germany) equilibrated with 0.1 M Tris-HCl at pH 8 and0.15 M NaCl that was previously degassed with argon. Complexes containing the Fd7Strep were eluted with 1.6 mL of elution buffer after washing with 2.5 mL of wash buffer, according to the manufacturer’s instructions, with the buffers being degassed with argon. A control experiment was run in parallel under the exact same conditions except that Fd7Strep was omitted and replaced by 100 µL of 0.1 M Tris-HCl at pH 8 and 0.15 M NaCl buffer. Eluted proteins from both columns were separated on a denaturing gel and stained with Coomassie Blue as well as on a BN gel (after dialysis on a Vivaspin concentrator (molecular mass cutoff 3000 Da) with 4 mL of 50 mM Tris-HCl at pH 8, 1 mM DTT, and 1 mM DTH, degassed with argon) for in-gel detection of PFOR and OGOR activities as described above.

### 2.9. Protein Identification and Proteomic Profiles by Mass Spectrometry

For global protein identification (proteomic profiles), two methods of protein extraction and digestion were used. The first method (“stacking method”) allowed for the preparation of soluble and membrane extracts by re-suspending *A. aeolicus* cells in 50 mM Tris-HCl at pH 7.6 with protease inhibitors (SIGMAFAST tablets). The cells were lysed with a cell disrupter as described above (paragraph 2.8) to obtain the total soluble extract (supernatant) and the membrane fraction (pellets re-suspended in 50 mM Tris-HCl at pH 7.6 to have a protein concentration of 10 mg mL^−1^). The membrane fraction was then solubilized with SDS at 5% (*w*/*v*) for 30 min at 37 °C. The total proteins present in the soluble extract and the membrane fraction were identified by shotgun proteomics after loading and migration of both samples (100 µg) on a denaturing stacking gel (5% acrylamide) for approximately 10 min at 25 mA and 250 V (migration was stopped when the dye front reached the limit between the stacking gel and the running gel). The protein band visible in the stacking gel after staining with Coomassie blue for each sample and containing theoretically the totality of proteins present in the extract was cut out from the gel and stored at −20 °C before LC-MS/MS analysis to identify proteins. This experiment was performed in duplicate. The second method of protein sample preparation (“S-Trap method”) used the S-Trap^TM^ Micro Spin Column (Protifi, Fairport, NY, USA) according to the manufacturer’s instructions. Before LC-MS/MS analysis, a total protein extract (soluble and membrane proteins) was obtained with 5% SDS and digested in the S-Trap^TM^ column. This experiment was performed in duplicate. In all cases, samples were digested with porcine trypsin (Promega, Charbonnières-les-Bains, France), and then analyzed with an ESI-Q-Exactive Plus Quadrupole-Orbitrap mass spectrometer (ThermoFisher Scientific, Illkirch-Graffenstaden, France) coupled to a nano-LC system (Ultimate 3000, Dionex, Sunnyvale, CA) equipped with an EASY-spray column (PepMaP TM RSLC, C18, 2 µm, 75 µm ID × 15 cm, Thermo Fisher Scientific, Illkirch-Graffenstaden, France). Tryptic peptides were separated by a two-step linear gradient from 6% to 40% of mobile phase B (0.1% (*v*/*v*) formic acid (FA)/80% (*v*/*v*) acetonitrile) in mobile phase A (0.1% (*v*/*v*) FA) for 52 min. For peptide ionization in the nanosource spray, the voltage was set at 1.9 kV and the capillary temperature at 275 °C. Top 10 Data Dependent workflow was used in a 400–1600 m/z range and a dynamic exclusion of 60 s. The spectra were processed by Proteome Discoverer software (ThermoFisher Scientific, version: 2.4.1.15) using the Sequest HT algorithm with the search following settings: *A. aeolicus* (taxonomy ID 63363 and subtaxonomies) extracted from Uniprot (3388 sequence entries, version 2020-12-16) plus the sequences of *A. aeolicus* Fd6 (NCBI protein ID: WP_024015104) and Fd7 (NCBI protein ID: WP_164930743.1) which were added manually; trypsin enzyme (maximum 2 missed cleavages); fixed modification: carbamidomethyl (Cys); variable modification: oxidation (Met); mass values specific for monoisotopic; precursor mass tolerance: ±10 ppm; fragment mass tolerance: ±0.02 Da. Peptide validation was based on a score threshold of maximum Delta Cn 0.05. If at least two unique peptide sequences containing more than six amino acids passed the high confidence filter, proteins were identified. An approximate estimation of the level of proteins in the extracts was based on the number of MS spectral counts (Peptide Spectral Match (PSM) counting) [27]. Data are available via ProteomeXchange with the identifier PXD040248.

## 3. Results

### 3.1. Carbon Fixation and Serine and Glycine Synthesis Pathways: Proteomic Profile of A. aeolicus Grown with H_2_ and Thiosulfate

**Figure 1:**
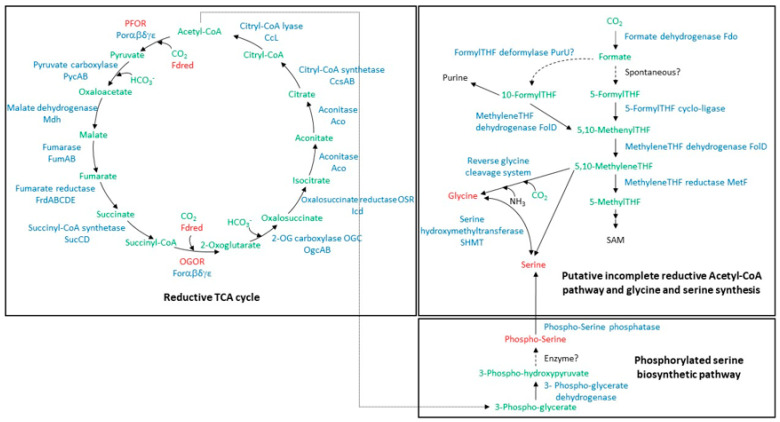
Putative pathways involved in carbon assimilation and serine biosynthesis in *A. aeolicus*. The rTCA cycle, the incomplete Wood-Ljungdahl pathway, and the phosphorylated serine synthesis pathway are represented [1,17]. A 10-Formyl-THF synthetase gene is missing in the genome and may be replaced by a Formyl-THF deformylase working in reverse [16]. Dotted arrows represent reactions that remain to be elucidated [1]. Enzymes are indicated in blue (except PFOR and OGOR, highlighted in red), and intermediates in green (except glycine, serine, and phospho-serine, highlighted in red). THF: tetrahydrofolate; SAM: S-Adenosyl Methionine; Fd and Fdred: ferredoxin and reduced ferredoxin.

Putative pathways of carbon assimilation and biosynthesis of the two amino acids glycine and serine are speculative in *A. aeolicus* and need to be further investigated. As described above, it was earlier suggested that two disjoint parallel CO_2_ fixation pathways might exist in *A. aeolicus*, namely the rTCA cycle and a possible linear folate-based pathway of CO_2_ reduction for glycine and serine biosynthesis [1,15] (Figure 1). With the aim of defining the protein expression profile of enzymes involved in these putative pathways, a shotgun proteomic analysis was conducted by LC-MS/MS on *A. aeolicus* protein extracts prepared using two different protocols. As the same trend was observed (approximate abundance of proteins followed by spectral counting), regardless of the approach used to prepare the samples, Table 1 shows only the results obtained with the “stacking method” on the total soluble fraction as well as the membrane fraction of the bacterium. Results for all experiments are reported in Table S1. Enzymes involved in the rTCA cycle (including five PFOR and five OGOR subunits) were fully identified and are among the most abundant in the soluble extract of *A. aeolicus*, as their PSM (peptide spectrum match) number, number of unique peptides, and sequence coverage are among the highest determined for the 800 proteins identified in this experiment (Table 1 and Table S1). In addition, Fd6 and Fd7, although very small in size, were both identified from the soluble content of *A. aeolicus* with a PSM number of 13 and 3 and a sequence coverage of 45 and 42%, respectively. A few years ago, a direct reductive biosynthesis from CO_2_, known as direct one-carbon reduction or reductive glycine pathway, was proposed for a number of organisms, including *A. aeolicus*, and was proposed to be widely distributed and the most likely ancestral pathway to the amino-acids glycine and serine [1,15,28,29,30]. In this pathway, CO_2_ is reduced to formate by a formate dehydrogenase working as a CO_2_ reductase, which is subsequently carried by the pterin cofactor tetrahydrofolate (THF). Glycine and serine are synthesized through the reverse of the glycine cleavage system (a lipoyl-based system) and serine hydroxymethyl transferase (Figure 1). We identified most of the proteins supposed to catalyze the steps of the incomplete Wood-Ljungdahl pathway and glycine cycle in the soluble content of *A. aeolicus*, whereas two subunits of the membranous three-subunit formate dehydrogenase were identified in the membrane fraction. The low PSM values obtained compared to proteins from the rTCA cycle (Table 1 and Table S1) suggest that these proteins are considerably less abundant in the cell. Moreover, the putative 5-FormylTHF cyclo-ligase was not detected at all, and the glycine cleavage system H proteins were hardly detected (Table 1 and Table S1). One possibility is that this route may not operate in *A. aeolicus*, at least under the growth conditions used in the present study, and that the corresponding enzymes that are identified by mass spectrometry may potentially be involved in other metabolic pathways. Glycine and serine could be synthesized as described in *H. thermophilus*, via a novel phosphoserine phosphatase, which is essential for serine anabolism in this bacterium [17]. The corresponding enzyme in *A. aeolicus* (accession number O67797; Locus tag Aq_1990; annotated as Phosphoglycerate mutase; 65% sequence identity with the *H. thermophilus* enzyme) was identified in the soluble content of the bacterium with 10 unique peptides and a sequence coverage of 56% (Table 1 and Table S1 and Figure 1).

In addition to enzymes related to carbon assimilation, the proteins Aq_866 (misannotated as NuoL2, accession number O67027) and Aq_863 (O67026) were also detected in the membrane fraction. These two proteins are probably part of the recently discovered membrane-bound heterodimeric complex DabAB that functions as an energy-coupled inorganic carbon pump in many prokaryotes [31]. This complex seems to be highly abundant in the membrane of *A. aeolicus*, as the PSM number of DabA is one of the highest (PSM number of 190, not shown). These proteomics data confirmed that *A. aeolicus* assimilates CO_2_ through the rTCA cycle [14]. The synthesis of serine and glycine may proceed via the phosphoserine route and may involve the novel phosphoserine phosphatase and the SHMT [17].

### 3.2. A. aeolicus Fd6 and Fd7 Are Low-Potential, Oxygen-Stable Ferredoxins

The rTCA cycle relies on reduced Fd (Figure 1). Two adjacent genes encoding two [4Fe-4S] Fds were previously identified in the genome of *A. aeolicus* [11] and could have a key role in carbon assimilation via the rTCA cycle. The proteins were purified from the soluble fraction of *A. aeolicus* and gave a yield of 2.5 and 1.5 mg, respectively, from 50 g of cells. Automated Edman degradation of 1 nmol of protein resulted in two distinct amino terminal sequences (MGLKVRVDQTCTACEL and MAKLKTMVDQETCTACEL), which correspond to the gene products of two ORF sequences previously detected and annotated as *fdx6* and *fdx7* [11]. The proteins were then identified as Fd6 (NCBI protein ID: WP_024015104, Locus Tag: Aq_1622a) and Fd7 (NCBI protein ID: WP_164930743.1, no. Locus Tag), respectively (Figure S1; [11]). Fd6 and Fd7 are 76 and 80 residues long, respectively, and show 76% identity to each other; the corresponding apoproteins’ mass spectra indicate a molecular mass of 8380 Da (Fd6) and 9130 Da (Fd7) (data not shown). The amino acid sequences contain a CX_2_CX_2_CX_n_CP motif (*n* = 47 and 48 in Fd6 and Fd7, respectively) that is typical of certain [4Fe-4S] proteins, such as ferredoxin I (FdI) from *Desulfovibrio africanus* and the one from *Bacillus thermoproteolyticus* [33]. A multiple sequence alignment of Fd6 and Fd7 with other characterized or putative Fds from other Aquificaceae containing a single cubane cluster (only Ht-Fd1 and Ht-Fd2 from *H. thermophilus* are characterized) shows that both *A. aeolicus* Fds contain an extra pair of cysteines (Figure S1). The presence of two additional cysteines in single cubane Fds is not unusual, as they are derived from a missing second [4Fe-4S] cluster and are often recruited for the formation of a S-S bond involved in thiol interconversion [34,35]. However, in Fd6 and Fd7, the two additional cysteines are not in positions resembling those of a typical S-S bridge. Thus, if these cysteines have a role, it will likely be structural, or, as observed for *A. aeolicus* [2Fe-2S] Fd1, they might contribute to protein thermostability [36]. However, this pair of cysteines does not occur in other Fds (except in the one from *Hydrogenothermus marinus*), even in closer homologs that are also thermophilic (Figure S1).

**Figure 2:**
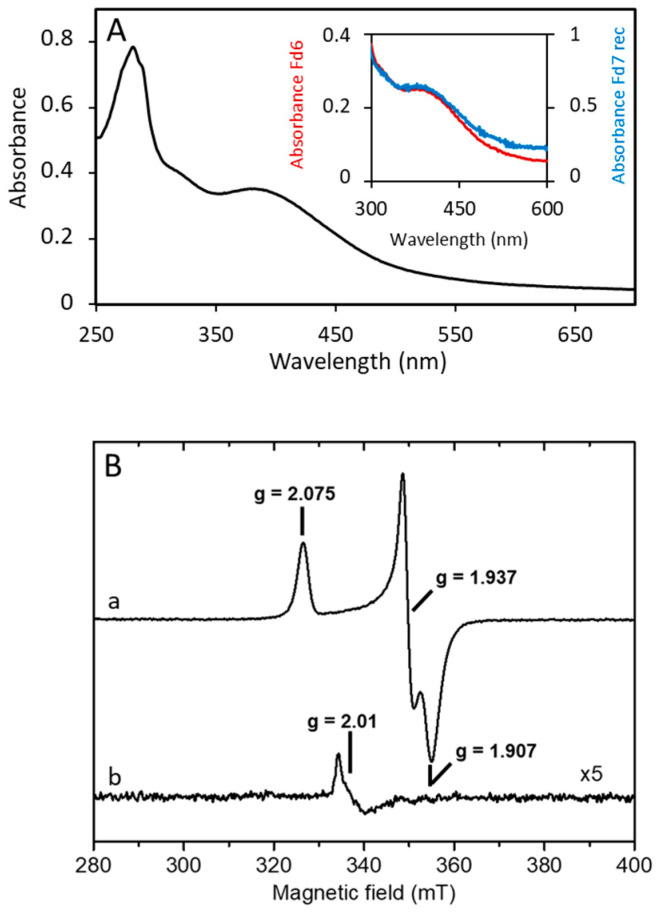
Spectroscopic properties of *A. aeolicus* ferredoxins. (**A**) UV-visible absorption spectrum of the “as purified” recombinant Fd7Strep. The inset shows a comparison of spectra for the Fd6 (red line) and Fd7Strep (blue line). They were purified in the presence of air and diluted in 0.1 M Tris-HCl at pH 8 and 0.15 M NaCl; (**B**) EPR spectra of the aerobically purified Fd7Strep in DTH-reduced (a) and as-purified (b) states. Experimental conditions: temperature, 15 K; microwave power, 1 mW at 9.481 GHz; modulation amplitude, 0.5 mT at 100 kHz.

For further experiments, we overexpressed Fd7 as a C-terminal Strep-tag II fusion protein. The yield was 12 mg of mature Fd7 from 4 L of *E. coli* culture. The UV-visible absorption spectra of both native Fd6 and recombinant Fd7 (Fd7Strep) display a broad absorption band centered at 380 nm (Figure 2A), which is consistent with the presence of one iron-sulfur cofactor. The spectra gave an A_390_/A_280_ ratio of 0.65 and 0.51 for Fd6 and Fd7Strep, respectively, which are similar to those for other purified Fds [37].

Considering the high sequence homology between Fd6 and Fd7 and the limited amount of Fd6, only Fd7Strep was investigated by EPR. At low temperature, aerobically purified Fd7Strep gave, after reduction by DTH, a rhombic spectrum with g-values of 2.075, 1.937, and 1.907 (Figure 2B, spectrum a). This spectrum broadened with relaxation above 20 K and disappeared at about 50 K. These properties are typical of a S = 1/2 [4Fe-4S]^1+^ cluster [38], and no other spectral features were observed at a low magnetic field, discarding the presence of higher spin states for the cluster [39]. Spin intensity measurements in non-saturating conditions yielded 1 ± 0.05 spin per molecule, which confirming the presence of one [4Fe-4S] center per Fd molecule. In the oxidized, as-prepared state of Fd7Strep, a very weak nearly isotropic signal at g = 2.01 characteristic of an oxidized [3Fe-4S]^1+^ cluster [38] and representing less than 0.02 spin/molecule could be detected (Figure 2B, spectrum b). As no differences in the UV-visible spectra and in the EPR spectra (Figure S2) have been observed when the Fd was purified aerobically or anaerobically, these results demonstrate the stability of Fd7Strep and of its [4Fe-4S] cluster in the presence of oxygen.

The redox potentials (E^0^) of native Fds, measured by direct electrochemistry using CV and SWV at 25 °C, were −440 +/− 10 mV for Fd6 and −460 +/− 10 mV for Fd7 (Figure S3). These potentials are among the most negative determined so far for single [4Fe-4S] Fds [12,40] and are consistent with the extremely low potential required to fulfill the energetic demand of the rTCA cycle.

### 3.3. Identification, Activity, and Oxygen-Sensitivity of A. aeolicus PFOR and OGOR

**Figure 3:**
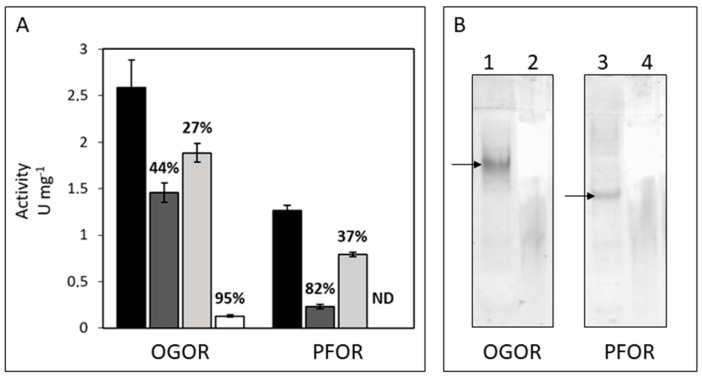
Activity of *A. aeolicus* OGOR and PFOR. (**A**) Activity of OGOR and PFOR from total soluble proteins under different extraction/assay conditions. Proteins were either extracted anaerobically (-O_2_, black and light grey columns) or under partially aerobic conditions (+O_2_, dark grey and white columns), and the assay was performed either in the presence of reducing agents (1 mM DTT and 1 mM DTH (+DTT(H)), black and dark grey columns), or in the absence of DTT and DTH (-DTT(H), light grey and white columns). Ten 50 µg aliquots of proteins were used in the -O_2_+DTT(H) and -O_2_-DTT(H) conditions, and 25–100 µg in the +O_2_+DTT(H) and +O_2_-DTT(H) conditions. The percentage values correspond to the decrease of the enzyme activity in the different extraction/assay conditions, with respect to the -O_2_+DTT(H) condition; ND = not detected; (**B**) In-gel enzyme activity staining of OGOR and PFOR. Either total soluble proteins (65 µg) or solubilized membrane proteins (100 µg) from *A. aeolicus* were separated on a 10% acrylamide native gel. (1) OGOR assay on soluble proteins; (2) OGOR assay on membrane proteins; (3) PFOR assay on soluble proteins; (4) PFOR assay on membrane proteins. Bands corresponding to activities were highlighted with arrows.

PFOR and OGOR, the two marker enzymes for CO_2_ fixation via the rTCA cycle, have never been characterized before in *A. aeolicus*. This bacterium possesses two gene clusters annotated as PFOR, but it can be predicted that the cluster *aq_1166-aq_1171a* encodes an OGOR enzyme [5,13]. We first confirmed that both activities are well measurable on the soluble protein fraction of the bacterium. Activities were measured in the oxidative direction (decarboxylation) using the low-redox potential electron acceptor MeV (E^0^ = −450 mV), which corresponds to the opposite direction of what is assumed to happen in vivo. The optimal temperature and optimal pH for both enzyme activities were found to be 80 °C and 9 (Figure S4). These enzymes are typically highly oxygen-sensitive, but as *A. aeolicus* is microaerophilic and possesses enzymes that are tolerant to oxygen [41], we performed experiments under different reducing/oxidizing conditions to determine if PFOR and OGOR make an exception. According to the general rule, both enzymes are oxygen-sensitive, with OGOR activity being decreased by 95% when the enzyme was extracted and measured in the presence of air, whereas PFOR activity was completely inhibited (Figure 3A). The activities were indeed maintained when the enzymes were extracted and stored under completely anaerobic conditions and partially restored in the presence of reducing agents like DTT and DTH.

Despite their high sequence and structural similarities, *A. aeolicus* PFOR and OGOR are two distinct enzymes, as are those of *H. thermophilus* [3,5]. The in-gel enzyme activities (decarboxylation) conducted on total soluble proteins separated by native-PAGE using MeV as an electron acceptor indeed show two distinct bands depending on the enzyme substrate specificities: pyruvate for PFOR and 2-oxoglutarate for OGOR (Figure 3B). Tandem mass spectrometry performed on the gel bands where the two enzyme activities were revealed confirmed the previous result by identifying PFOR and OGOR subunits (Table 2; Figure S5). Both enzymes are composed of five subunits named FORαβγδε and PORαβγδε, respectively, for OGOR and PFOR. All 2-oxoacid:Fd oxidoreductases maintain typical features regardless of their structural composition [3]. Thus, some conserved regions with specific functions can also be identified in *A. aeolicus* OGOR and PFOR. The ε subunits, which correspond to the δ subunit in four-subunit 2-oxoacid oxidoreductases, are Fd-like proteins that arbor two [4Fe-4S] cluster-binding sites most probably responsible for the efficient electron transfer to/from an external electron acceptor/donor, and were consequently annotated as Fd2 (FORε) and Fd3 (PORε) [42]. The β subunits contain the conserved GDGx_4_ID domain, which is responsible for binding the TPP cofactor, and four additional conserved cysteines, including an atypical CX_2_C motif, which are putatively involved in the coordination of a third [4Fe-4S] cluster as suggested for *D. africanus* PFOR [43]. PORδ and FORδ are found exclusively in five-subunit type 2-oxoacid:Fd oxidoreductases from Aquificaceae [5] and conserve high sequence similarity with the β subunit of carbon monoxide dehydrogenase/acetyl-CoA synthase ε subunit (CODH/ACS) from methanogenic archaea and acetogenic bacteria performing carbon fixation via the Wood–Ljungdahl pathway [44]. However, this enzyme looks to be incomplete in *A. aeolicus* since no ACS genes have been found in the genome. Consequently, its function and, especially, why there are two copies located within the OGOR and PFOR gene clusters is intriguing. The *A. aeolicus* PFOR gene cluster also harbors one additional open reading frame named *aq_1194* [5], which codes for an uncharacterized protein (accession number O67253) that has homology with Uma2 family endonucleases (Figure S5). This protein, which was hardly or not detected in the total extract or soluble fraction of the bacterium in our proteomic analyses (data not shown), was not identified by mass spectrometry in the PFOR activity gel band (Table 2), suggesting that it is not part of the enzyme complex.

### 3.4. Fd6 and Fd7 Are Involved in the Reconstructed rTCA Cycle

**Figure 4:**
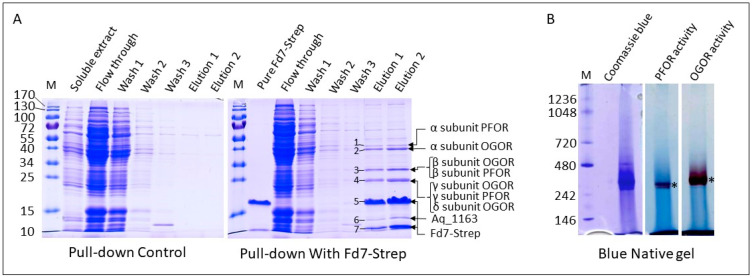
Pull-down assay using Fd7Strep. (**A**) Proteins from the various fractions of the pull-down assays (control on the left and pull-down on the right) were resolved by a 15% denaturing gel and stained with Coomassie Blue. Molecular weight markers are indicated on the left in kDa (lane M). Proteins in the seven major bands (lanes elution), identified by mass spectrometry, are indicated on the right (see Table S2 for details of their identification). The following volumes of fractions were loaded in each lane (from lane 2 to lane 8): 0.5 µL (4.5 µg), 1 µL, 2 µL, 10 µL, 20 µL, 35 µL (7 µg), 35 µL (17 µg) for the pull-down, and 0.5 µL, 1 µL, 2 µL, 10 µL, 20 µL, 35 µL (2 µg), 35 µL (2.8 µg) for the control experiment; (**B**) Proteins from the elution fraction 2 were separated on a 3–12% Blue Native gel. 5.8 µg of proteins were loaded in each lane and stained with Coomassie blue or PFOR, and OGOR activities were detected in-gel. The bands corresponding to the activity were marked with a *. See Table S3 for details on the identification of proteins from these bands. Molecular weight markers are indicated on the left in kDa (lane M).

To demonstrate whether a physical interaction occurs between Fd7Strep and either PFOR or OGOR, pull-down assays were performed using the Strep-tagged recombinant Fd7 as a bait for the soluble extract of *A. aeolicus*. A control experiment was performed in the same conditions but without the Fd7Strep. Proteins captured and eluted from the Strep-Tactin resin were detected on a denaturing gel stained with Coomassie blue (Figure 4A). Seven major bands were cut out from the gel, and the proteins were identified by mass spectrometry (Figure 4A; see Table S2 for details on protein identification). The interacting proteins were also separated on a Blue Native gel (Figure 4B). The detection of the in-gel PFOR and OGOR activities (at roughly 300 kDa) and the identification of all subunits of the two enzymes in the bands (Table S3) confirm that Fd7 forms a stable complex with both. Stable Fd-partner complexes were also maintained in pull-down assays in *Thermococcus kodakarensis* [45]. On the contrary, the complex between PFOR and FdI from *D. africanus* shows a low binding affinity typical of transient complex formation [46]. It seems that the stability of Fd-protein complexes is variable and depends, among other factors, on the redox state and the presence of substrates. It should be noted that for OGOR, in addition to the five Forαβγδε subunits, two proteins annotated as uncharacterized proteins, Aq_1163 and reverse gyrase 2, whose genes are adjacent to those encoding the OGOR subunits (Figure S5), were captured in the pull-down experiment and identified by proteomics (Tables S2 and S3). The small protein Aq_1163 has no sequence homology with proteins of known functions, whereas reverse gyrase 2 is predicted to be an ATP-dependent DNA topoisomerase. The fact that these two proteins co-migrate on the BN gel with OGOR may suggest that all these proteins form one or several complexes of the same size. Aq_1163 and the reverse gyrase 2 were also identified (data not shown) in the band of OGOR activity detected in the total soluble extract in the native gel (Figure 3B, lane 1).

To show whether the two Fds are able to exchange electrons with PFOR and OGOR, we used a coupled assay in which Fd reduction by PFOR or OGOR was indirectly followed by Fd re-oxidation by the artificial electron acceptor MTZ (E^0^ = −486 mV). According to the results obtained for the native enzymes, we produced two enriched fractions of PFOR and OGOR under partially aerobic conditions and decided to perform all tests in the presence of the reducing agents DTT and DTH in order to preserve most of their activity. Both enzymes were obtained from the soluble fraction of *A. aeolicus*: most of the PFOR activity was obtained after a DEAE affinity chromatography step that gave 37 U of enzyme (0.33 U/mg); for OGOR, a second step by HTP affinity chromatography was performed and gave 117 U (1.71 U/mg) (Table 3). In the coupled assay, OGOR was able to efficiently reduce MTZ via both Fd6 and recombinant Fd7Strep, with very similar specific activities (0.30 U/mg with Fd7Strep and 0.26 U/mg with Fd6) when the OGOR-enriched fraction protein concentration was kept constant at 25 µg/mL (Table 3; Figure S6). OGOR was not active towards MTZ when the Fd was omitted. OGOR specific activity with Fds is in the same range as previously found for *H. thermophilus* PFOR and Ht-Fd1 [47]. The same could not be observed for the *A. aeolicus* PFOR-enriched fraction since PFOR-specific activity could not be detected in the presence of either Fd6 or Fd7Strep using MTZ as an electron acceptor, very likely because its basal activity was only 0.33 U/mg using MeV as an electron acceptor, a value that is about 5-fold lower than that of OGOR (Table 3). As shown in Figure 3A, this might be due to the much higher sensitivity of this enzyme to oxygen, which resulted in a lower purification fold. In spite of this, considering the high similarity between PFOR and OGOR, it is very likely that the two enzymes behave in the same manner in vivo. Overall, these results demonstrate that Fd7 strongly interacts with both PFOR and OGOR and that an electron transfer takes place between them.

## 4. Discussion

In this work, we aim at describing carbon assimilation in the obligate chemolithoautotroph *A. aeolicus* via the identification of the key enzymes involved in the rTCA cycle: PFOR and OGOR, as well as their low potential electron donors, Fds. We confirmed that the two homologous gene clusters (*aq_1166*-*aq_1171a* and *aq_1192a*-*aq_1200*; Figure S5) do indeed encode one PFOR and one OGOR, and that Fd2 and Fd3 correspond, respectively, to the ε subunits of OGOR and PFOR. These enzyme complexes are most likely formed of at least five subunits and exist in a dimeric form (αβγδε)_2_, since their molecular masses are estimated to be about 250 to 300 kDa from the BN gel, like the enzymes present in *H. thermophilus* [3,5,7]. Currently, these types of pentameric enzymes have only been characterized biochemically in the Aquificaceae (i.e., *A. aeolicus* and *H. thermophilus)*. The uncharacterized protein Aq_1163, of about 15 kDa and encoded by a gene directly flanking the OGOR gene cluster (Figure S5), could potentially interact with the OGOR complex because (1) this protein is captured from the total soluble extract using Fd7Strep (note that Aq_1163 is abundant in the Strep-Tactin eluted protein fraction as evidenced on the SDS gel in Figure 4A), (2) it co-migrates with OGOR under non-denaturing condition (Figure 3 and Figure 4), and (3) the apparent molecular mass of the OGOR activity band on the various gels is slightly larger than that of the PFOR activity band while their respective theoretical molecular mass is nearly identical, which may indicate the presence of additional protein(s) in the OGOR complex compared to PFOR. A homolog to *aq_1163* was found only in *Hydrogenivirga* sp. 128-5-R1-1 and in *Hydrogenivirga caldilitoris*, both members of the Aquificales and phylogenetically related to *A. aeolicus*. The two putatively corresponding proteins share 36–38% sequence identity with Aq_1163, and their genes are not adjacent to the OGOR gene cluster in *Hydrogenivirga* species. As this protein does not appear to be conserved, it would suggest that *A. aeolicus* possesses a unique and specific OGOR complex, not present in most of the other related species. Given its theoretical molecular mass (134 kDa), the reverse gyrase 2, identified as well in the pull-down assay and whose gene is adjacent to aq_1163 and the OGOR gene cluster, is presumably not directly associated with the OGOR complex. Further studies are needed to validate this result and determine the role of these proteins. As in *H. thermophilus* or in the bacteria *Nitrospira marina* [48] and *Thermovibrio ammonificans* [16], both enzyme complexes were abundant in the *A. aeolicus* proteome, confirming their significant role in central carbon metabolism. Other possible 5-subunit OGOR/PFOR, for which structural data are lacking, were also identified in genomes of aerobic or micro-aerophilic bacteria that fix CO_2_ via the rTCA cycle, like *Leptospirillum* [49], *Nitrospina gracilis* [50], *Nitrospira* [48], *Sulfurovum* [3], or *Manganitrophus* genera [51], and were proposed to be possibly more O_2_-tolerant than other enzymes of the same family [9,52].

Our attempt to elucidate the pathway also showed that the *A. aeolicus* CO_2_-fixing machinery involves the two homologous, tandemly arranged [4Fe-4S] Fd6 and Fd7, which are likely natural redox partners of PFOR and OGOR and serve as low potential electron donors for the reductive carboxylation reactions performed by these two enzymes, as in its close relative *H. thermophilus* [6,8]. Fd6 and Fd7 redox potentials, together with the one determined for Ht-Fd1 (−485 mV at pH 7 and 23 °C), are the lowest currently determined for Fds containing one [4Fe-4S] center [12,40]. This is consistent with the involvement of these Fds in the reductive carboxylation of acetyl-CoA and succinyl-CoA, an energetically challenging process [2,53,54]. Despite the additional two cysteine residues in Fd6 and Fd7 compared to those of *H. thermophilus*, there is no significant impact on the properties of the [Fe-S] center. It is intriguing to have two Fds that are highly similar (76% identity and roughly the same redox potential) in *A. aeolicus*, raising the question of their respective functions. In *H. thermophilus* TK-6, Ht-Fd1 and Ht-Fd2 present only 42% sequence identity to each other, yet they are both employed by the PFOR and OGOR of this bacterium in carboxylating as well as decarboxylating reactions in vitro [6,11]. Results obtained from in vivo protein-protein interactions in *T. kodakarensis* revealed that the OGOR is specific to Fd-2 from this archaeon while the PFOR uses all three Fds present in this heterotrophic micro-organism [45]. We have shown that both Fd6 and Fd7 are well produced in *A. aeolicus* under the growth conditions used. Sequence comparisons indicate that Ht-Fd1 is closer to *A. aeolicus* Fd6 and Fd7 (63–65% identity) than Ht-Fd2 is (only 36–37% identity). Two adjacent genes encoding two Fds are found in species of the family Aquificaceae (*Aquifex*, *Hydrogenivirga*, *Thermocrinis,* and *Hydrogenobacter*), whereas only one Fd appears to be present in members of the family Hydrogenothermaceae (Figure S1).

To be completely fulfilled, the pathway also requires the presence of enzymes capable of reducing the Fds in vivo, a process that has not been clarified yet in most of the chemolithoautotrophic microorganisms performing the rTCA cycle [1,2,54]. As Fds are low-potential redox proteins, the reduction reaction requires a strong reductant. Since *A. aeolicus* grows using hydrogen and/or reduced sulfur compounds as the energy substrate, the electrons used to maintain the reduced Fd pool in vivo likely originate from one of these sources. However, they all provide electrons with a redox potential higher than that of Fd6 or Fd7, meaning that this reaction may not be favorable. In general, when hydrogen is the electron source, reduction of low-potential Fd is achieved either by a membrane-bound energy-converting hydrogenase at the expense of the electrochemical membrane potential or by cytosolic or cytosol-oriented hydrogenases that use the mechanism of flavin-based electron bifurcation [19,55]. It was hypothesized that *Thermovibrio ammonificans*, which is part of the phylum *Aquificae* and relies on the rTCA cycle, can achieve Fd reduction by cytosolic Group 3 hydrogenases via electron bifurcation and a Group 4 membrane-bound Ech hydrogenase [16]. A survey of the genomic sequence of *A. aeolicus* that we carried out revealed that the genes coding for these different enzymatic systems are not found in this bacterium [16,55,56]. In spite of this, genes coding for three [NiFe] hydrogenases occur in *A. aeolicus* [26,54]. Hydrogenase (Hase) I, a well-characterized, high-redox potential [41], periplasmic and membrane-bound, respiratory H_2_-uptake Group 1d enzyme [57], could not be considered as a possible reductant for Fds, which are cytoplasmic proteins. Assays using purified Hase I confirmed that Fd7Strep is not reduced by this enzyme in the presence of hydrogen (data not shown). In the same way, neither of the two Fds could be reduced by the Hyn hydrogenase from *D. fructosovorans* that has [4Fe-4S] clusters with a redox potential of −340 mV [58] (data not shown), confirming previous experiments conducted with *H. thermophilus* Fds [5,8,12]. On the other hand, cytosolic *A. aeolicus* Hase III was hypothesized to be a potential candidate for the reduction of Fd [26,54]. This scheme of electron transfer might also occur in *H. thermophilus* since a cytoplasmic [NiFe] hydrogenase called Hup2 and highly similar to *A. aeolicus* Hase III is encoded by genes expected to be transcribed constitutively [59]. This enzyme belongs to Group 2d of [NiFe] hydrogenases, whose function is unresolved but could have a regulatory function [57]. This putative role is in agreement with the difficulty of detecting this enzyme in *A. aeolicus* extracts (by our shotgun proteomic approach and by western blot using a specific antibody; data not shown), suggesting that it is hardly involved in Fd reduction. The potential role of the membrane-bound *A. aeolicus* hydrogenase II (Group 1e of [NiFe] hydrogenase, [57]) in the reduction of low-potential Fds is completely unknown. Reducing equivalents derived from the sulfur compound oxidation may also be used to reduce the Fds required for carbon assimilation. Various enzymes involved in sulfur metabolism have been characterized in *A. aeolicus*, including a heterodisulfide reductase (Hdr)-like complex [60] that may be considered a potential candidate to play a role as direct redox partners of Fds. Hdr complexes that use the mechanism of electron bifurcation are known as Fd-reducing enzymes [55]. In *H. thermophilus* TK-6, genes for Ht-Fd1 and Ht-Fd2 are located in between genes for succinyl-CoA synthetase subunits and those for the Hdr-like system, which could suggest a functional link between these proteins. However, it was recently proposed that the Hdr-like complex from sulfur-oxidizing prokaryotes may not be able to perform electron bifurcation [61]. The supply of low-potential reducing equivalents in the form of Fd that drive carbon fixation is still unresolved in bacteria employing the rTCA.

In addition to the rTCA cycle, for which the full set of enzymes was identified in the proteome, it has been proposed that *A. aeolicus* synthesizes some biomass components by using an incomplete form of the Wood-Ljungdahl pathway and the reductive glycine pathway [1,15]. It was later suggested that an autotrophic bacterium from the Deltaproteobacteria assimilates CO_2_ and formate via the reductive glycine pathway as its sole carbon fixation pathway [29]. Our proteomic data indicate that most enzymes putatively involved in this carbon fixation pathway are retrieved from the *A. aeolicus* proteome, although they are clearly under-represented compared to those involved in the rTCA cycle (Table 1), which would suggest that the largest portion of the carbon is fixed by the rTCA pathway. The same trend was found by proteomic analyses in the deep-branching autotrophic bacterium *Thermovibrio ammonificans,* for which it was hypothesized that the reductive acetyl-CoA pathway is used as an additional or alternative pathway to fix CO_2_ [16]. Various elements must be taken into consideration regarding the possibility of such a potentially active reductive CO_2_ pathway in *A. aeolicus*. (i) The first point to consider is the conversion of CO_2_ to formate, which is typically catalyzed by a cytoplasmic NAD(P)-dependent CO_2_-reducing formate dehydrogenase [29,62,63]. It was recently proposed that reduction of CO_2_ to formate during autotrophic growth of *Desulfovibrio desulfuricans* is performed by a periplasmic trimeric and soluble molybdenum-containing formate dehydrogenase working with a cytochrome *c* as redox partner [30]. In *A. aeolicus*, the only formate dehydrogenase so far detected (encoded by *fdoGHI*), although still uncharacterized, is thought to be a periplasmic membrane-bound heterotrimeric enzyme (αβγ)_3_ proposed to operate in the direction of oxidation of formate to CO_2_ and reduction of the quinone pool in other bacteria [29,62,63]. The existence of this putatively incomplete reductive Acetyl-CoA pathway in *A. aeolicus* would imply reverse activity of this enzyme or involvement of a yet-to-be discovered enzyme. (ii) Another point is that the 5-formyl-THF cyclo-ligase, putatively involved in the reductive acetyl-CoA pathway, is not detected in the soluble fraction of *A. aeolicus,* and the involvement of a formyl-THF deformylase as an alternative enzyme that would function in reverse in the formation of 10-formyl-THF is speculative (although it is identified in cells) [16,64]. In consequence, the binding of formate to THF remains elusive (Figure 1). (iii) The synthesis of glycine in this carbon fixation pathway putatively uses the glycine cleavage system operating in reverse. The proteomic profile that we established allowed us to detect the presence of the members of this enzymatic system in *A. aeolicus*. However, in *H. thermophilus*, this system does not appear to be operative, as no activity was detected in the cell extract of the bacterium [17]. This may also be the case in *A. aeolicus*, which could synthesize glycine from serine via the phospho-serine phosphatase (PspA), experimentally proven to be essential for serine synthesis in *H. thermophilus*, and the serine hydroxymethyltransferase (SHMT), both identified in the soluble fraction of *A. aeolicus* (Figure 1). Due to the fact that only a few bacteria have been described to possess two carbon fixation pathways [16,65], it is critical to determine whether the putative partial Wood-Ljungdahl pathway is a true CO_2_ assimilation pathway and whether this pathway and the rTCA cycle both operate at the same time or are differentially activated in *A aeolicus*. More broadly, the interconnection between different CO_2_ assimilation pathways remains to be explored.

## Figures and Tables

**Table 1:** Tandem mass spectrometry identification of enzymes putatively involved in carbon assimilation in *A. aeolicus* (rTCA cycle, incomplete Wood-Ljungdahl pathway, and glycine and serine biosynthesis pathways).

Enzymes	Substrate → Product		Subunit/ Protein	Accession ^1^	Locus Tag	Gene	Mw ^2^	PSM ^3^	Cov ^4^	Pep ^5^
REVERSE TCA CYCLE										
Pyruvate: ferredoxin oxidoreductase (PFOR)	Acetyl-CoA → Pyruvate		Porα	O67254	aq_1195	*forA1*	45.1	178	63	20
			Porβ	O67255	aq_1196	*forB1*	32.2	44	55	14
			Porγ	O67256	aq_1200	*forG1*	26.7	84	72	14
			Porε (Fdx3)	O67251	aq_1192A	*forD1*	9	11	85	5
			Porδ	O67252	aq_1192	*aq_1192*	21.3	43	66	10
PEP synthase	Pyruvate → Phosphoenolpyruvate		PpsA	O67899	aq_2142	*ppsA*	96.4	220	64	59
Pyruvate carboxylase (PYC)	Pyruvate → Oxaloacetate		PycA	O67544	aq_1614	*oadA*	70.4	108	55	35
			PycB	O67449	aq_1470	*accC2*	53.7	64	55	29
Malate dehydrogenase (MDH)	Oxaloacetate → Malate		Mdh1	O67655	aq_1782	*mdh1*	36.7	85	71	19
			Mdh2	O67581	aq_1665	*mdh2*	36.7	9	27	7
Fumarase (FUM)	Malate → Fumarate		FumA	O67654	aq_1780	*fumB*	30.9	25	38	12
			FumB	O67590	aq_1679	*fumX*	20.4	21	61	11
Fumarate reductase (FRD)	Fumarate → Succinate		FrdA	O66855	aq_594	*frdA*	63.9	105	69	38
			FrdB	O66828	aq_553	*frdB1*	27	4	16	3
			FrdC	O66518	aq_116	*aq_116*	59.5	21	28	12
			FrdD	O67007	aq_835	*nox*	53.3	26	59	18
			FrdE	O66481	aq_067	*dmsB2*	19.4	5	18	3
Succinyl-CoA synthetase (SUC)	Succinate → Succinyl-CoA		SucC	O67546	aq_1620	*sucC*	42.2	97	69	30
			SucD	O67547	aq_1622	*sucD2*	32.2	105	58	12
2-Oxoglutarate: ferredoxin oxidoreductase (OGOR)	Succinyl-CoA → 2-Oxoglutarate		Forα	O67229	aq_1167	*forA2*	44.7	253	72	26
			Forβ	O67230	aq_1168	*forB2*	32.6	76	69	20
			Forγ	O67231	aq_1169	*forG2*	25.5	89	92	18
			Forε (Fdx2)	O67232	aq_1171	*forD2*	9	30	91	7
			Forδ	O67228	aq_1166	*aq-1166*	27.6	149	84	21
2-Oxoglutarate carboxylase (OGC)	2-Oxoglutarate → Oxalosuccinate		OgcA (CfiA)	O67484	aq_1520	*pycA*	73.6	472	74	56
			OgcB (CfiB)	O67483	aq_1517	*pycB*	52.8	244	74	33
Oxalosuccinate reductase (OSR)	Oxalosuccinate → Isocitrate		Icd	O67480	aq_1512	*icd*	46.9	54	48	23
Aconitase	Isocitrate → Aconitate → Citrate		Aco	O67656	aq_1784	*aco*	72.5	133	67	38
Citryl-CoA synthetase (CCS)	Citrate → Citryl-CoA		CcsA	O67330	aq_1306	*sucC1*	48.3	231	77	35
			CcsB	O67729	aq_1888	*sucD1*	39.8	164	78	30
Citryl-CoA lyase (CCL)	Citryl-CoA → Acetyl-CoA + Oxaloacetate		Ccl	O66541	aq_150	*gltA*	29.1	95	80	21
PUTATIVE INCOMPLETE REDUCTIVE ACETYL-CoA PATHWAY and GLYCINE AND SERINE SYNTHESIS										
Formate dehydrogenase	CO_2_ → Formate		FdoI FdoH FdoG	O67148 O67147 O67146	aq_1049 aq_1046 aq_1039	*fdoI* *fdoH* *fdoG*	24.3 33.4 114.5	- 10 17	- 24 14	- 6 12
Formyltetrahydrofolate deformylase	Formate → 10-formylTHF		PurU	O67681	aq_1818	*purU*	32.8	8	41	8
5-Formyltetrahydrofolate cyclo-ligase	5-formylTHF → 5,10-methenylTHF		MTHFS	O67621	aq_1731	*aq_1731*	21.2	-	-	-
Methylenetetrahydrofolate dehydrogenase	10-formylTHF → 5,10-methenylTHF → 5,10-methyleneTHF		FolD	O67736	aq_1898	*folD*	31.9	18	37	9
5,10-methylenetetrahydrofolate reductase	5,10-methyleneTHF → 5-methylTHF		MetF	O67422	aq_1429	*metF*	33.9	19	49	13
Dihydrolipoyl dehydrogenase	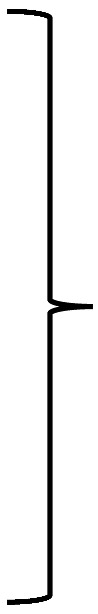		GcvL/Lpd	O66945	aq_736	*lpdA*	51.6	54	72	25
Aminomethyl transferase			GcvT	O67441	aq_1458	*gcvT*	40.3	27	46	17
Glycine dehydrogenase (decarboxylating) subunit 1		Reversible glycine cleavage system	GcsP2	O67193	aq_1109	*gcvPA*	49.7	32	40	19
Glycine dehydrogenase (decarboxylating) subunit 2			GcsP1	O67740	aq_1903	*gcvPB*	55	30	45	18
Glycine cleavage system H protein 1		CO_2_ +NH_3_ + 5,10-methyleneTHF → Glycine + THF	GcsH	O67151	aq_1052	*gcvH1*	16.2	-	-	-
Glycine cleavage system H protein 2			GcsH	O67573	aq_1657	*gcvH2*	18.2	3	29	3
Glycine cleavage system H protein 3			GcsH	O67080	aq_944	*gcvH3*	18.1	-	-	-
Glycine cleavage system H protein 4			GcsH	O67192	aq_1108	*gcvH4*	19.6	-	-	-
Serine hydroxymethyltransferase	Glycine + 5,10-methyleneTHF → Serine + THF		SHMT	O66776	aq_479	*glyA*	47.4	63	46	21
SERINE AND GLYCINE SYNTHESIS via phosphorylated serine										
Phospho-serine phosphatase	Phospho-serine → Serine		PspA	O67797	aq_1990	*pgmA*	24.4	17	56	10
Serine hydroxymethyltransferase	Serine + THF → Glycine + 5,10-methyleneTHF		SHMT	O66776	aq_479	*glyA*	47.4	63	46	21

Proteins were identified from the total soluble extract (except for the formate dehydrogenase, which is a membrane-bound enzyme identified in the detergent-solubilized membrane fraction) of the bacteria grown with hydrogen and thiosulfate in autotrophic conditions. Results reported in this table were obtained after protein preparation with the “stacking method”, as described in the Material and Methods section, and correspond to one of two independent experiments. Results for all replicates and those obtained with the “S-Trap method” are reported in Table S1. The protein GcvH5 (renamed LbpA2 [32]), accession number O66720; locus tag aq_402), was identified by mass spectrometry in the soluble extract but not included in the table because it was proposed to be involved in the sulfur oxidation pathway and not in the glycine cleavage system. ^1^ Accession: accession number in the UniProt database. ^2^ Mw: theoretical molecular weight of the identified protein in kDa. ^3^ PSM: peptide spectrum match number (given by the algorithm and corresponding to the total number of identified peptide sequences for the protein, including those redundantly identified).^4^ Cov: percent protein sequence coverage by the matching peptides. ^5^ Pep: number of distinct peptides matching the protein sequence and unique to this protein. -: not identified by mass spectrometry.

**Table 2:** Tandem mass spectrometry identification of OGOR and PFOR subunits from in-gel activity-stained bands. Bands were cut out from the native gel (Figure 3B) (lane 1 and lane 3). Only subunits of both enzymes are reported in the table.

	Description	Subunit	Accession ^1^	Locus Tag	Gene	Mw ^2^	PSM ^3^	Cov ^4^	Pep ^5^
OGOR in-gel activity staining	Alpha subunit OGOR	For*α*	O67229	aq_1167	*forA2*	42.7	62	58	17
	Beta subunit OGOR	Forβ	O67230	aq_1168	*forB2*	32.6	30	26	8
	Gamma subunit OGOR	Forγ	O67231	aq_1169	*forG2*	25.5	25	84	12
	Epsilon subunit OGOR	Forε	O67232	aq_1171	*forD2 (fdx2)*	9	4	43	3
	Delta subunit OGOR	Forδ	O67228	aq_1166	*aq_1166*	27.6	23	57	10
PFOR in-gel activity staining	Alpha subunit PFOR	For*α*	O67254	aq_1195	*forA1*	45.1	113	66	19
	Beta subunit PFOR	Porβ	O67255	aq_1196	*forB1*	32.2	28	47	10
	Gamma subunit PFOR	Porγ	O67256	aq_1200	*forG1*	26.6	20	63	10
	Epsilon subunit PFOR	Porε	O67251	aq_1192a	*forD1 (fdx3)*	9.1	7	26	3
	Delta subunit PFOR	Porδ	O67252	aq_1192	*aq_1192*	21.3	17	61	7
	Uma2 domain-containing protein		O67253	aq_1194	*aq_1194*	20.9	-	-	-

^1^ Accession: accession number in the UniProt database. ^2^ Mw: theoretical molecular weight of the identified protein in kDa. ^3^ PSM: peptide spectrum match number (given by the algorithm corresponding to the total number of identified peptide sequences for the protein, including those redundantly identified). ^4^ Cov: percent protein sequence coverage by the matching peptides. ^5^ Pep: number of distinct peptides matching the protein sequence and unique to this protein. -: not identified by mass spectrometry.

**Table 3:** Activity of OGOR and PFOR with different electron acceptors.

	Electron Acceptor ^2^	Activity (U mg^−1^)
**OGOR ^1^**	MeV	1.71 ± 0.10
	MTZ	ND
	MTZ + Fd6	0.30 ± 0.01
	MTZ + Fd7	0.26 ± 0.02
**PFOR ^1^**	MeV	0.33 ± 0.02
	MTZ	ND
	MTZ + Fd6	ND
	MTZ + Fd7	ND

^1^ 20–50 µg of protein from the enriched OGOR fraction, 50–100 µg of protein from the enriched PFOR were used. ^2^ MeV (−450 mV) was used at 5 mM, MTZ (−486 mV) at 0.1 mM, and Fd (native Fd6 or Fd7Strep) at 10 µM.

## Data Availability

The mass spectrometry raw data have been deposited to the ProteomeXchange Consortium [67] (http://proteomecentral.proteomexchange.org) via the PRIDE [68] partner repository, with the dataset identifier PXD040248, accessed on 18 February 2023.

## Supplementary Materials

The following supporting information can be downloaded at: https://www.mdpi.com/article/10.3390/life13030627/s1, Figure S1: Multiple sequence alignment of the *A. aeolicus* Fd6 and Fd7 and Aquificales single [4Fe-4S] cluster Fds; Figure S2: Spectroscopic properties of *A. aeolicus* Fd7Strep; Figure S3: SWV signal for Fd6 and Fd7; Figure S4: Dependence on pH and temperature of PFOR and OGOR activities; Figure S5: PFOR and OGOR gene clusters of *A. aeolicus*; Figure S6: *A. aeolicus* Fd7Strep-linked reduction of MTZ, using OGOR, Table S1: Tandem mass spectrometry identification of proteins involved in the rTCA cycle, the putative linear folate-based pathway of CO_2_ reduction, or the phosphorylated serine pathway for glycine and serine biosynthesis; Table S2: Tandem mass spectrometry identification of proteins occurring in the seven major bands of the denaturing gel shown in Figure 4A (elution from the pull-down assay), Table S3: Tandem mass spectrometry identification of proteins occurring in the bands revealed by PFOR and OGOR activity on the BN gel [66].
