# Supplementary material for: Carbon Fixation in the Chemolithoautotrophic Bacterium Aquifex aeolicus Involves Two Low-Potential Ferredoxins as Partners of the PFOR and OGOR Enzymes
Source: Life (Basel). 2023 Feb 23;13(3):627. doi: 10.3390/life13030627 (PMC10052474; doi:10.3390/life13030627)
Supplement: Supplementary file 1 [file life-13-00627-s001.zip › life-2221381-supplementary updated/Supplementary Figures_revised.pptx]

## Slide 1
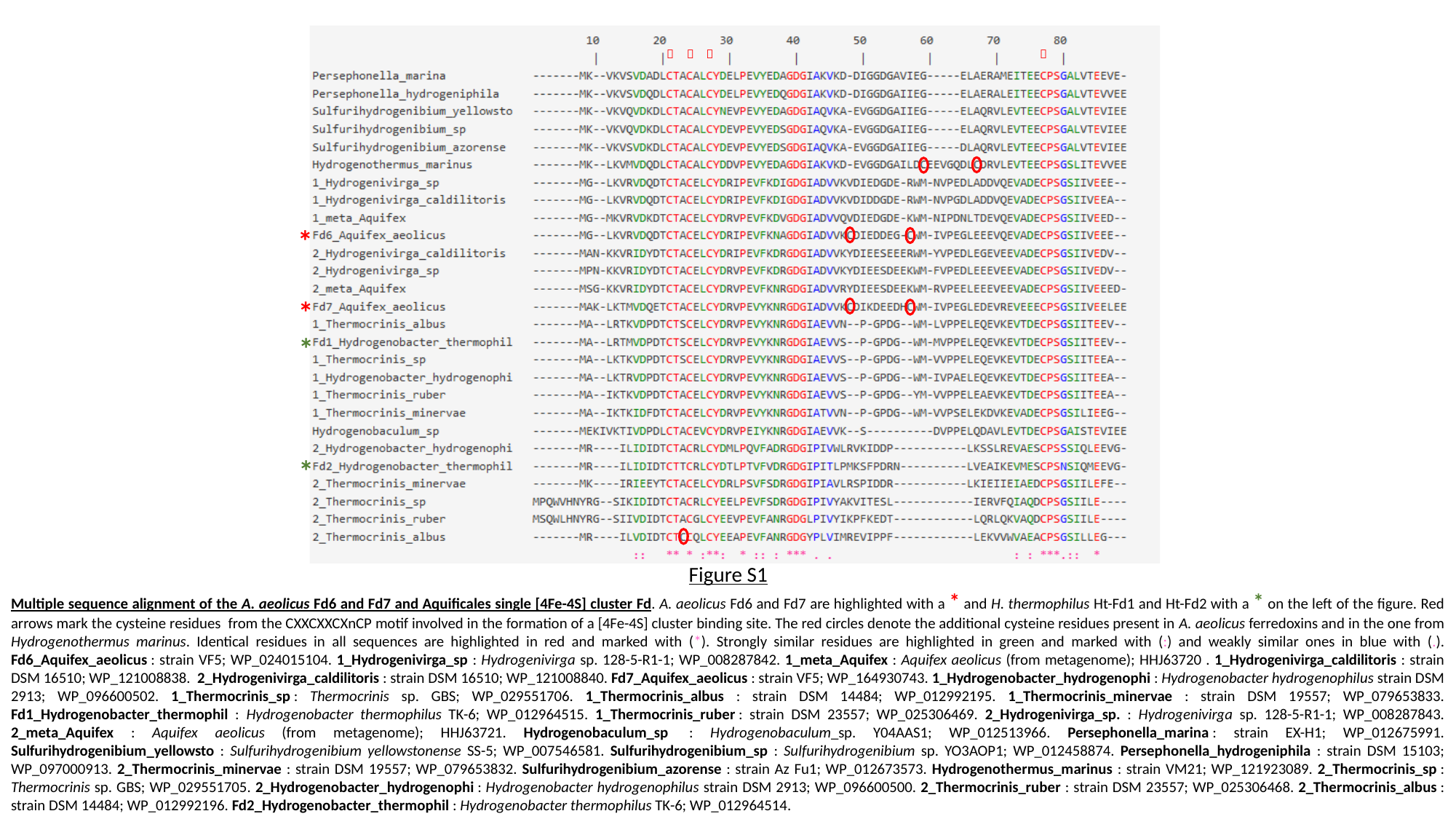





Figure S1
*
*
*
*
Multiple sequence alignment of the A. aeolicus Fd6 and Fd7 and Aquificales single [4Fe-4S] cluster Fd. A. aeolicus Fd6 and Fd7 are highlighted with a * and H. thermophilus Ht-Fd1 and Ht-Fd2 with a * on the left of the figure. Red arrows mark the cysteine residues from the CXXCXXCXnCP motif involved in the formation of a [4Fe-4S] cluster binding site. The red circles denote the additional cysteine residues present in A. aeolicus ferredoxins and in the one from Hydrogenothermus marinus. Identical residues in all sequences are highlighted in red and marked with (*). Strongly similar residues are highlighted in green and marked with (:) and weakly similar ones in blue with (.). Fd6_Aquifex_aeolicus : strain VF5; WP_024015104. 1_Hydrogenivirga_sp : Hydrogenivirga sp. 128-5-R1-1; WP_008287842. 1_meta_Aquifex : Aquifex aeolicus (from metagenome); HHJ63720 . 1_Hydrogenivirga_caldilitoris : strain DSM 16510; WP_121008838. 2_Hydrogenivirga_caldilitoris : strain DSM 16510; WP_121008840. Fd7_Aquifex_aeolicus : strain VF5; WP_164930743. 1_Hydrogenobacter_hydrogenophi : Hydrogenobacter hydrogenophilus strain DSM 2913; WP_096600502. 1_Thermocrinis_sp : Thermocrinis sp. GBS; WP_029551706. 1_Thermocrinis_albus : strain DSM 14484; WP_012992195. 1_Thermocrinis_minervae : strain DSM 19557; WP_079653833. Fd1_Hydrogenobacter_thermophil : Hydrogenobacter thermophilus TK-6; WP_012964515. 1_Thermocrinis_ruber : strain DSM 23557; WP_025306469. 2_Hydrogenivirga_sp. : Hydrogenivirga sp. 128-5-R1-1; WP_008287843. 2_meta_Aquifex : Aquifex aeolicus (from metagenome); HHJ63721. Hydrogenobaculum_sp  : Hydrogenobaculum_sp. Y04AAS1; WP_012513966. Persephonella_marina : strain EX-H1; WP_012675991. Sulfurihydrogenibium_yellowsto : Sulfurihydrogenibium yellowstonense SS-5; WP_007546581. Sulfurihydrogenibium_sp : Sulfurihydrogenibium sp. YO3AOP1; WP_012458874. Persephonella_hydrogeniphila : strain DSM 15103; WP_097000913. 2_Thermocrinis_minervae : strain DSM 19557; WP_079653832. Sulfurihydrogenibium_azorense : strain Az Fu1; WP_012673573. Hydrogenothermus_marinus : strain VM21; WP_121923089. 2_Thermocrinis_sp : Thermocrinis sp. GBS; WP_029551705. 2_Hydrogenobacter_hydrogenophi : Hydrogenobacter hydrogenophilus strain DSM 2913; WP_096600500. 2_Thermocrinis_ruber : strain DSM 23557; WP_025306468. 2_Thermocrinis_albus : strain DSM 14484; WP_012992196. Fd2_Hydrogenobacter_thermophil : Hydrogenobacter thermophilus TK-6; WP_012964514.

## Slide 2
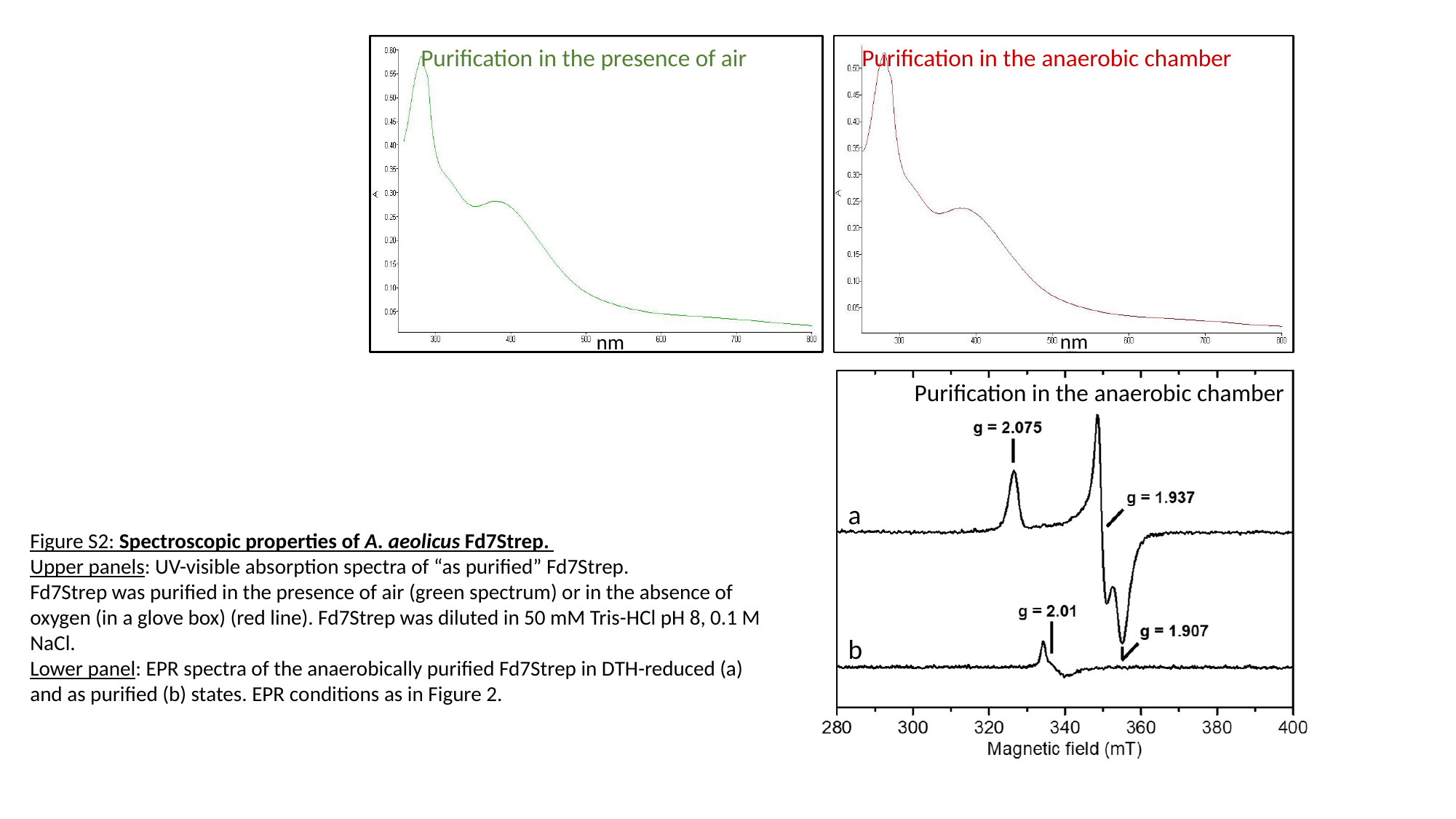

Purification in the anaerobic chamber
Purification in the presence of air
nm
nm
Purification in the anaerobic chamber
a
b
Figure S2: Spectroscopic properties of A. aeolicus Fd7Strep.
Upper panels: UV-visible absorption spectra of “as purified” Fd7Strep.
Fd7Strep was purified in the presence of air (green spectrum) or in the absence of oxygen (in a glove box) (red line). Fd7Strep was diluted in 50 mM Tris-HCl pH 8, 0.1 M NaCl.
Lower panel: EPR spectra of the anaerobically purified Fd7Strep in DTH-reduced (a) and as purified (b) states. EPR conditions as in Figure 2.

## Slide 3
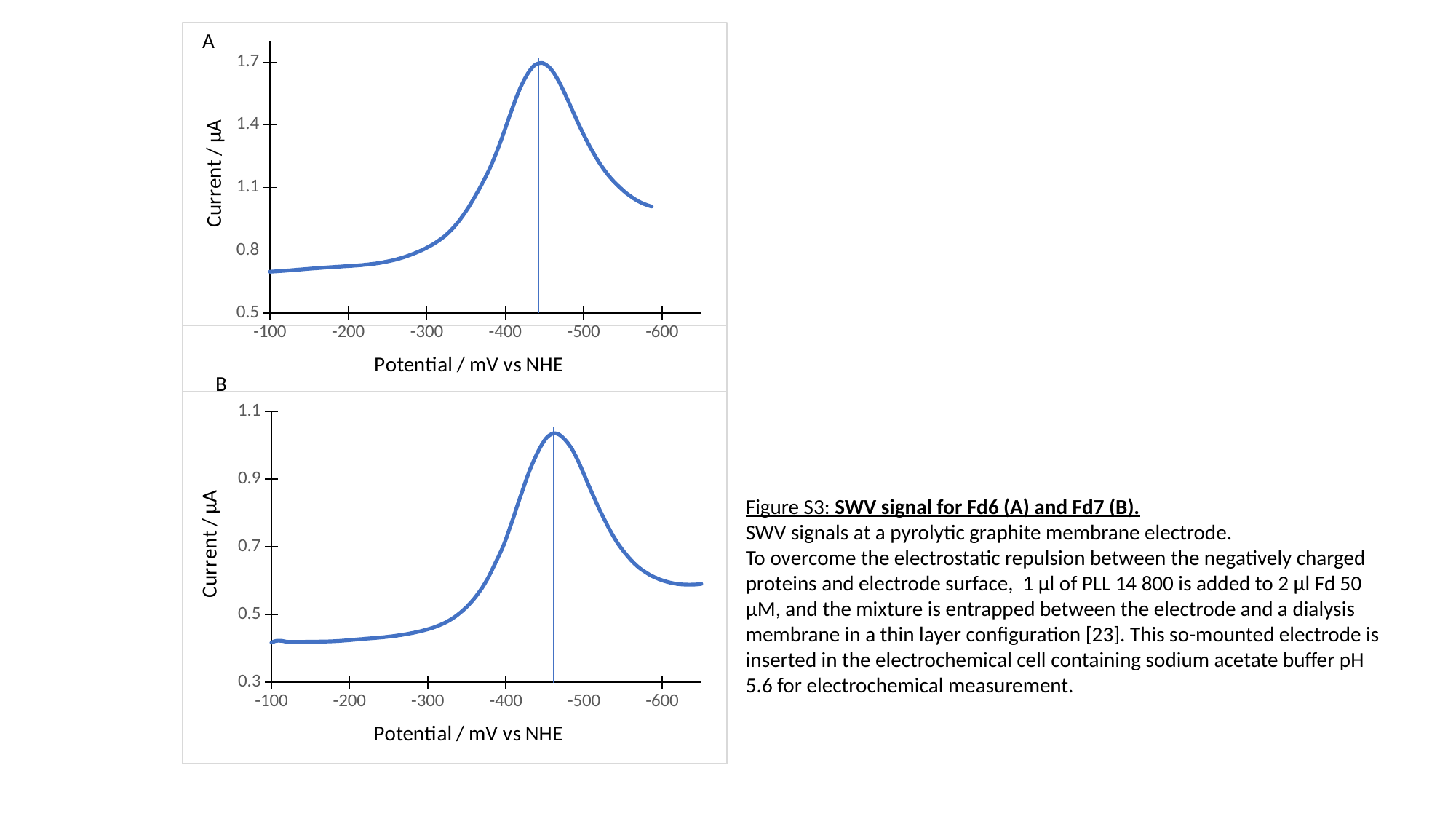

### Chart
| Category | FD I current |
|---|---|A
### Chart
| Category | FD II current |
|---|---|B
Figure S3: SWV signal for Fd6 (A) and Fd7 (B).
SWV signals at a pyrolytic graphite membrane electrode.
To overcome the electrostatic repulsion between the negatively charged proteins and electrode surface, 1 µl of PLL 14 800 is added to 2 µl Fd 50 µM, and the mixture is entrapped between the electrode and a dialysis membrane in a thin layer configuration [23]. This so-mounted electrode is inserted in the electrochemical cell containing sodium acetate buffer pH 5.6 for electrochemical measurement.

## Slide 4
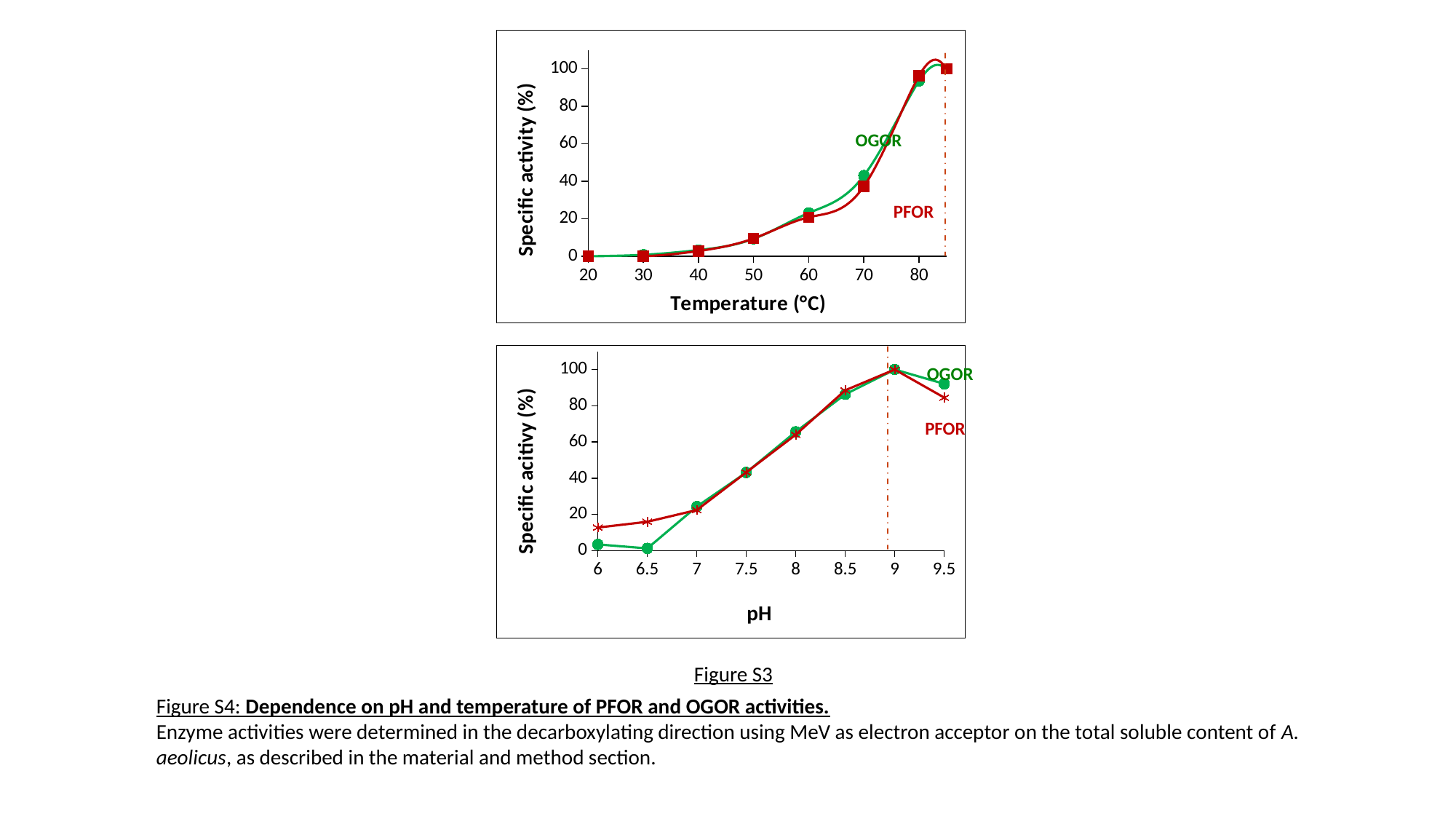

### Chart
| Category | | |
|---|---|---|OGOR
PFOR
### Chart
| Category | pH | |
|---|---|---|OGOR
PFOR
Figure S3
Figure S4: Dependence on pH and temperature of PFOR and OGOR activities.
Enzyme activities were determined in the decarboxylating direction using MeV as electron acceptor on the total soluble content of A. aeolicus, as described in the material and method section.

## Slide 5
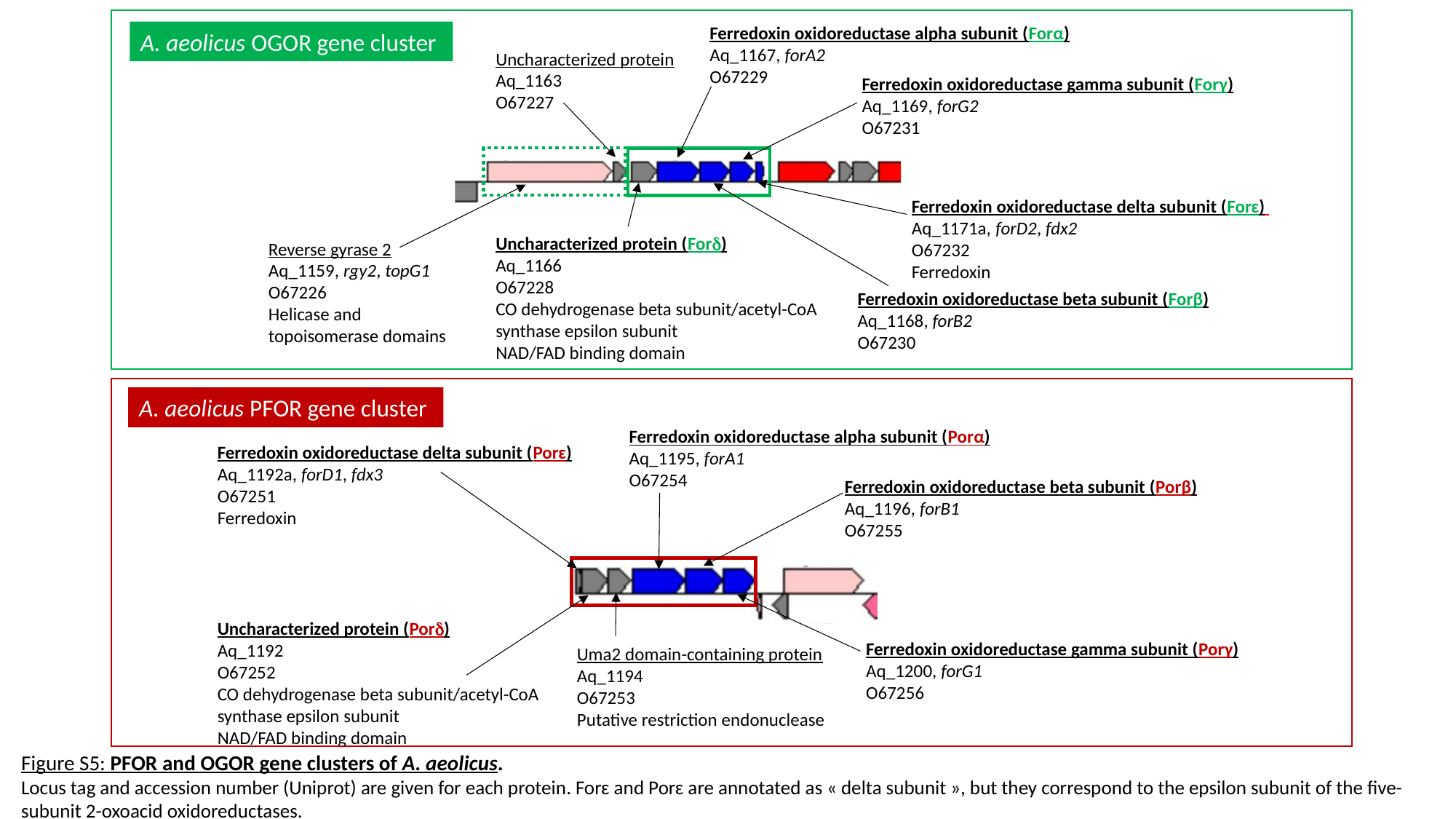

Ferredoxin oxidoreductase alpha subunit (Forα)
Aq_1167, forA2
O67229
A. aeolicus OGOR gene cluster
Uncharacterized protein
Aq_1163
O67227
Ferredoxin oxidoreductase gamma subunit (Forγ)
Aq_1169, forG2
O67231
Ferredoxin oxidoreductase delta subunit (Forɛ)
Aq_1171a, forD2, fdx2
O67232
Ferredoxin
Uncharacterized protein (Forδ)
Aq_1166
O67228
CO dehydrogenase beta subunit/acetyl-CoA synthase epsilon subunit
NAD/FAD binding domain
Reverse gyrase 2
Aq_1159, rgy2, topG1
O67226
Helicase and topoisomerase domains
Ferredoxin oxidoreductase beta subunit (Forβ)
Aq_1168, forB2
O67230
A. aeolicus PFOR gene cluster
Ferredoxin oxidoreductase alpha subunit (Porα)
Aq_1195, forA1
O67254
Ferredoxin oxidoreductase delta subunit (Porɛ)
Aq_1192a, forD1, fdx3
O67251
Ferredoxin
Ferredoxin oxidoreductase beta subunit (Porβ)
Aq_1196, forB1
O67255
Uncharacterized protein (Porδ)
Aq_1192
O67252
CO dehydrogenase beta subunit/acetyl-CoA synthase epsilon subunit
NAD/FAD binding domain
Ferredoxin oxidoreductase gamma subunit (Porγ)
Aq_1200, forG1
O67256
Uma2 domain-containing protein
Aq_1194
O67253
Putative restriction endonuclease
Figure S5: PFOR and OGOR gene clusters of A. aeolicus.
Locus tag and accession number (Uniprot) are given for each protein. Forɛ and Porɛ are annotated as « delta subunit », but they correspond to the epsilon subunit of the five-subunit 2-oxoacid oxidoreductases.

## Slide 6
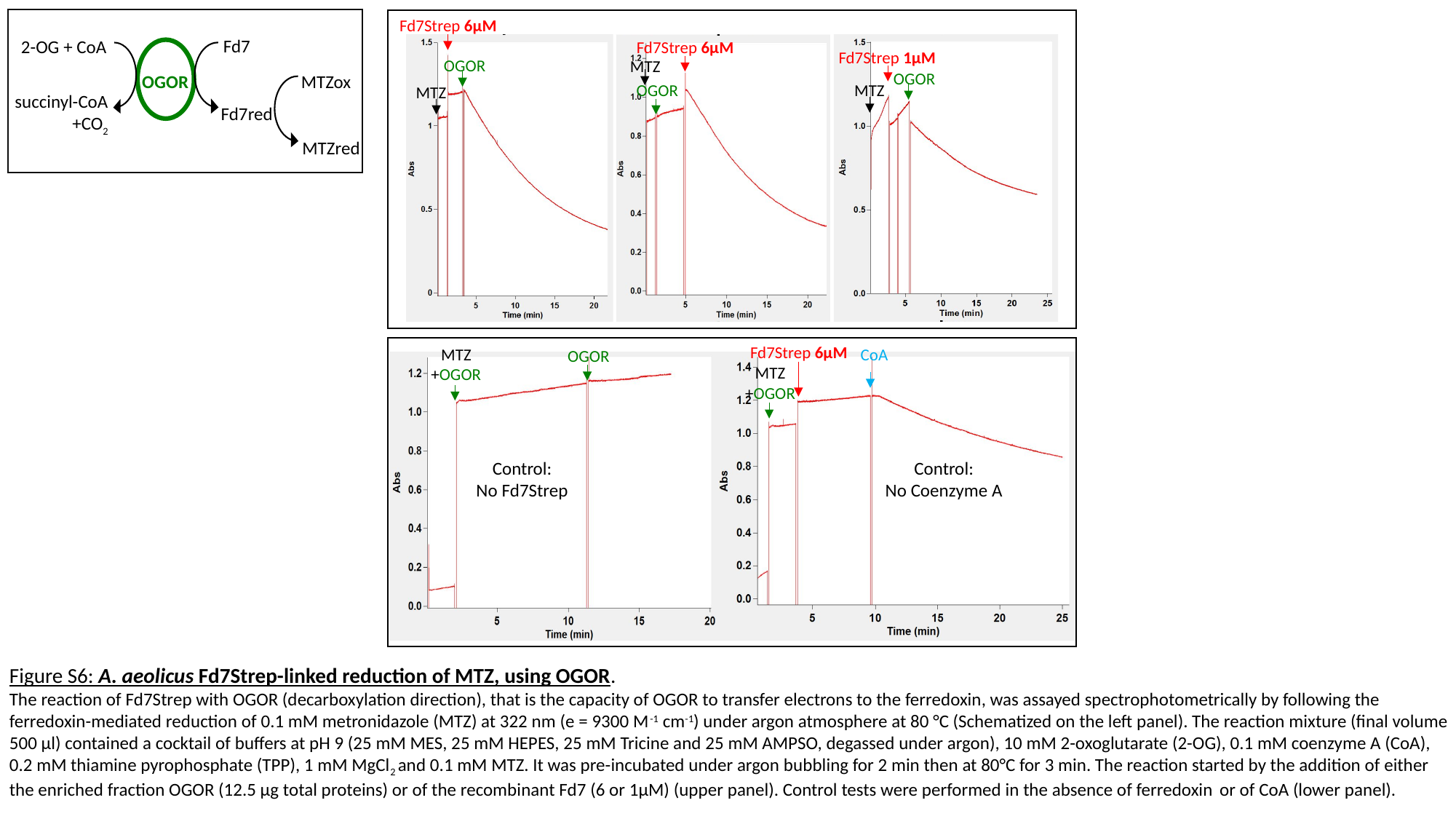

Fd7
2-OG + CoA
OGOR
MTZox
succinyl-CoA +CO2
Fd7red
MTZred
Fd7Strep 6µM
Fd7Strep 6µM
Fd7Strep 1µM
OGOR
MTZ
OGOR
MTZ
OGOR
MTZ
Fd7Strep 6µM
MTZ
+OGOR
CoA
OGOR
MTZ
+OGOR
Control:
No Fd7Strep
Control:
No Coenzyme A
Figure S6: A. aeolicus Fd7Strep-linked reduction of MTZ, using OGOR.
The reaction of Fd7Strep with OGOR (decarboxylation direction), that is the capacity of OGOR to transfer electrons to the ferredoxin, was assayed spectrophotometrically by following the ferredoxin-mediated reduction of 0.1 mM metronidazole (MTZ) at 322 nm (e = 9300 M-1 cm-1) under argon atmosphere at 80 °C (Schematized on the left panel). The reaction mixture (final volume 500 µl) contained a cocktail of buffers at pH 9 (25 mM MES, 25 mM HEPES, 25 mM Tricine and 25 mM AMPSO, degassed under argon), 10 mM 2-oxoglutarate (2-OG), 0.1 mM coenzyme A (CoA), 0.2 mM thiamine pyrophosphate (TPP), 1 mM MgCl2 and 0.1 mM MTZ. It was pre-incubated under argon bubbling for 2 min then at 80°C for 3 min. The reaction started by the addition of either the enriched fraction OGOR (12.5 µg total proteins) or of the recombinant Fd7 (6 or 1µM) (upper panel). Control tests were performed in the absence of ferredoxin or of CoA (lower panel).
