# Supplementary material for: Carbon Fixation in the Chemolithoautotrophic Bacterium Aquifex aeolicus Involves Two Low-Potential Ferredoxins as Partners of the PFOR and OGOR Enzymes
Source: Life (Basel). 2023 Feb 23;13(3):627. doi: 10.3390/life13030627 (PMC10052474; doi:10.3390/life13030627)
Supplement: Supplementary file 1 [file life-13-00627-s001.zip › life-2221381-supplementary updated/Supplementary tables_revised.pdf]

					“Stacking” 1			“Stacking“ 2			“S-Trap” 1			“S-Trap” 2		
**ENZYMES**	**SUBUNIT**	**ACCESSION**	**LOCUS TAG**	**GENE**	**PSM**	**COV**	**PEP**	**PSM**	**COV**	**PEP**	**PSM**	**COV**	**PEP**	**PSM**	**COV**	**PEP**
**REVERSE TCA CYCLE**																
**PFOR**	Porα	O67254	aq_1195	*forA1*	178	63	20	88	49	14	186	55	15	216	57	16
	Porβ	O67255	aq_1196	*forB1*	44	55	14	38	34	6	63	46	10	46	16	6
	Porγ	O67256	aq_1200	*forG1*	84	72	14	62	54	16	50	62	10	86	68	10
	Porε (Fdx3)	O67251	aq_1192A	*forD1*	11	85	5	-	-	-	40	67	5	7	15	2
	Porδ	O67252	aq_1192	*aq_1192*	43	66	10	21	40	6	26	54	6	46	56	8
**PEP synthase**	PpsA	O67899	aq_2142	*ppsA*	220	64	59	181	55	47	11	49	34	163	48	36
**Pyruvate carboxylase (PYC)**	PycA	O67544	aq_1614	*oadA*	108	55	35	89	48	30	80	53	21	72	38	16
	PycB	O67449	aq_1470	*accC2*	64	55	29	48	43	22	23	38	15	42	45	18
**Malate dehydrogenase (MDH)**	Mdh1	O67655	aq_1782	*mdh1*	85	71	19	62	50	14	83	56	11	119	51	12
	Mdh2	O67581	aq_1665	*mdh2*	9	27	7	7	16	4	11	23	7	8	7	2
**Fumarase (FUM)**	FumA	O67654	aq_1780	*fumB*	25	38	12	23	29	11	44	64	14	41	48	13
	FumB	O67590	aq_1679	*fumX*	21	61	11	38	64	10	16	45	7	32	57	9
**Fumarate reductase (FRD)**	FrdA	O66855	aq_594	*frdA*	105	69	38	70	53	29	57	54	26	58	46	21
	FrdB	O66828	aq_553	*frdB1*	4	16	3	4	17	3	5	24	4	6	17	3
	FrdC	O66518	aq_116	*aq_116*	21	28	12	17	25	11	14	24	9	17	20	10
	FrdD	O67007	aq_835	*nox*	26	59	18	16	30	10	6	23	6	15	37	11
	FrdE	O66481	aq_067	*dmsB2*	5	18	3	2	22	2	3	25	3	-	-	-
**Succinyl-CoA synthetase (SUC)**	SucC	O67546	aq_1620	*sucC*	97	69	30	71	55	19	100	63	22	83	53	18
	SucD	O67547	aq_1622	*sucD2*	105	58	12	91	60	14	69	60	14	82	54	12
**OGOR**	Forα	O67229	aq_1167	*forA2*	253	72	26	175	68	23	283	67	20	202	56	16
	Forβ	O67230	aq_1168	*forB2*	76	69	20	59	61	17	107	55	14	111	39	10
	Forγ	O67231	aq_1169	*forG2*	89	92	18	77	66	14	80	79	14	93	90	15
	Forε (Fdx2)	O67232	aq_1171	*forD2*	30	91	7	14	63	4	36	58	4	15	26	2
	Forδ	O67228	aq_1166	*aq-1166*	149	84	21	94	63	17	88	75	15	74	52	12
**2-Oxoglutarate carboxylase (OGC)**	OgcA (CfiA)	O67484	aq_1520	*pycA*	472	74	56	308	65	47	268	65	40	319	62	34
	OgcB (CfiB)	O67483	aq_1517	*pycB*	244	74	33	116	62	26	106	63	20	124	58	21
**Oxalosuccinate reductase (OSR)**	Icd	O67480	aq_1512	*icd*	54	48	23	73	49	22	95	51	15	100	52	16
**Aconitase**	Aco	O67656	aq_1784	*aco*	133	67	38	113	41	24	118	59	27	119	47	23
**Citryl-CoA synthetase (CCS)**	CcsA	O67330	aq_1306	*sucC1*	231	77	35	149	61	27	194	61	25	208	56	23
	CcsB	O67729	aq_1888	*sucD1*	164	78	30	154	79	23	137	64	17	139	57	17
**Citryl-CoA lyase (CCL)**	Ccl	O66541	aq_150	*gltA*	95	80	21	62	50	14	80	63	13	93	63	14
**PUTATIVE INCOMPLETE REDUCTIVE ACETYL-CoA PATHWAY and GLYCINE AND SERINE SYNTHESIS**																
**Formate dehydrogenase**	FdoI	O67148	aq_1049	*fdoI*	-	-	-	-	-	-	-	-	-	-	-	-
	FdoH	O67147	aq_1046	*fdoH*	10	24	6	7	21	4	6	30	5	-	-	-
	FdoG	O67146	aq_1039	*fdoG*	17	14	12	27	24	17	16	21	13	3	4	3
**FormylTHF deformylase**	PurU	O67681	aq_1818	*purU*	8	41	8	14	45	10	6	29	5	10	31	6
**5-FormylTHF cyclo-ligase**	MTHFS	O67621	aq_1731	*aq_1731*	-	-	-	-	-	-	-	-	-	-	-	-
**MethyleneTHF dehydrogenase**	FolD	O67736	aq_1898	*folD*	18	37	9	16	32	7	9	29	6	15	23	7
**5,10-methyleneTHF reductase**	MetF	O67422	aq_1429	*metF*	19	49	13	15	30	6	10	31	7	6	19	4
**Dihydrolipoyl dehydrogenase**	GcvL/Lpd	O66945	aq_736	*lpdA*	54	72	25	85	61	28	48	57	18	82	59	21
**Aminomethyl transferase**	GcvT	O67441	aq_1458	*gcvT*	27	46	17	20	32	11	11	26	7	14	24	7
**Glycine dehydrogenase (decarboxylating) subunit 1**	GcsP2	O67193	aq_1109	*gcvPA*	32	40	19	20	29	12	10	19	7	16	24	9
**Glycine dehydrogenase (decarboxylating) subunit 2**	GcsP1	O67740	aq_1903	*gcvPB*	30	45	18	31	34	15	27	41	17	29	33	15
**Glycine cleavage system H protein 1**	GcsH	O67151	aq_1052	*gcvH1*	-	-	-	-	-	-	2	26	2	4	10	2

**Glycine cleavage system H protein 2**	GcsH	O67573	aq_1657	*gcvH2*	3	29	3	-	-	-	19	51	6	18	37	4
**Glycine cleavage system H protein 3**	GcsH	O67080	aq_944	*gcvH3*	-	-	-	-	-	-	-	-	-	4	32	3
**Glycine cleavage system H protein 4**	GcsH	O67192	aq_1108	*gcvH4*	-	-	-	-	-	-	-	-	-	2	25	2
**Serine hydroxymethyltransferase**	SHMT	O66776	aq_479	*glyA*	63	46	21	53	37	14	27	34	11	38	36	13
**SERINE AND GLYCINE SYNTHESIS via phosphorylated serine**																
**Phospho-serine phosphatase**	PspA	O67797	aq_1990	*pgmA*	17	56	10	14	40	6	9	32	6	16	30	6
**Serine hydroxymethyltransferase**	SHMT	O66776	aq_479	*glyA*	63	46	21	53	37	14	27	34	11	38	36	13

Table S1 : Tandem mass spectrometry identification of proteins involved in the rTCA cycle, the putative linear folate-based pathway of CO_2 reduction or the phosphorylated serine pathway for glycine and serine biosynthesis

Results reported in this table were obtained after protein preparation with the “stacking method” or the “S-Trap method” as described in the Material and Method section. Results are from two independent experiments. 800, 730, 962 and 1046 proteins were identified in “Stacking” 1, “Stacking” 2, “S-Trap” 1 and “S-Trap” 2, respectively. Accession: accession number in UniProt database. PSM: peptide spectrum match number (given by the algorithm corresponding to the total number of identified peptide sequences for the protein, including those redundantly identified. Protein abundance approximation). COV: percent protein sequence coverage by the matching peptides. PEP: number of distinct peptides matching to protein sequence and unique to this protein. The protein GcvH5 (renamed LbpA2 (Cao, 2018), accession number O66720; locus tag aq_402) was identified by mass spectrometry in the soluble extract but not included in the table because it was proposed to be involved in sulfur oxidation pathway and not in the glycine cleavage system.

Reference :

Cao X, Koch T, Steffens L, Finkensieper J, Zigann R, Cronan JE, Dahl C. Lipoate-binding proteins and specific lipoate-protein ligases in microbial sulfur oxidation reveal an atpyical role for an old cofactor. Elife. 2018;7:e37439. doi: 10.7554/eLife.37439.

Band Figure 4A	Description	Subunit	Accession	Locus Tag	Gene	MW	PSM	COV	PEP
1	Alpha subunit PFOR	Porα	O67254	aq_1195	*forA1*	45.1	351	59	14
2	Alpha subunit OGOR	Forα	O67229	aq_1167	*forA2*	42.7	119	46	16
3	Beta subunit OGOR	Forβ	O67230	aq_1168	*forB2*	32.6	484	53	16
	Beta subunit PFOR	Porβ	O67255	aq_1196	*forB1*	32.2	111	35	10
4	Gamma subunit OGOR	Forγ	O67231	aq_1169	*forG2*	25.5	457	89	17
	Gamma subunit PFOR	Porγ	O67256	aq_1200	*forG1*	26.7	69	54	13
	Delta subunit OGOR	Forδ	O67228	aq_1166	*aq_1166*	27.6	239	73	17
5	Fd7-Streptag		-	-	*fdx7*	11	40	72	5
6	Uncharacterized protein		O67227	aq_1163	*aq_1163*	14.9	217	65	9
7	Fd7-Streptag		-	-	*fdx7*	11	42	34	4

Table S2 : Tandem mass spectrometry identification of proteins occurring in the seven major bands of the denaturing gel shown in Figure 4A (elution from the pulldown assay).

Only subunits of PFOR (in red), proteins related to the OGOR (in green) and the Fd7 are reported in the table. Accession: accession number in UniProt database. MW: theoretical molecular weight of the identified protein in kDa. PSM: peptide spectrum match number (given by the algorithm corresponding to the total number of identified peptide sequences for the protein, including those redundantly identified. Protein abundance approximation). COV: percent protein sequence coverage by the matching peptides. PEP: number of distinct peptides matching to protein sequence and unique to this protein.

Description	Subunit	Accession	Locus Tag	Gene	MW	PSM	COV	PEP
**OGOR activity band**								
Alpha subunit OGOR	Forα	O67229	aq_1167	*forA2*	42.7	848	75	32
Gamma subunit OGOR	Forγ	O67231	aq_1169	*forG2*	25.5	623	93	20
Beta subunit OGOR	Forβ	O67230	aq_1168	*forB2*	32.6	397	69	21
Delta subunit OGOR	Forδ	O67228	aq_1166	*aq_1166*	27.6	346	79	20
Alpha subunit PFOR	Porα	O67254	aq_1195	*forA1*	45.1	292	68	19
Beta subunit PFOR	Porβ	O67255	aq_1196	*forB1*	32.2	130	40	13
Epsilon subunit OGOR	Forε	O67232	aq_1171	*forD2*	9	128	91	10
Gamma subunit PFOR	Porγ	O67256	aq_1200	*forG1*	26.7	102	42	13
Delta subunit PFOR	Porδ	O67252	aq_1192	*aq_1192*	21.3	63	45	7
Uncharacterized protein		O67227	aq_1163	*aq_1163*	14.9	49	69	9
Glutamate synthase small subunit		O67845	aq_2064	*gltD*	52.9	24	45	18
Uncharacterized protein		O66853	aq_591	*aq_591*	39.2	14	39	11
Reverse gyrase 2		O67226	aq_1159	*rgy2*	134.5	13	10	11
Reverse gyrase 1		O67037	aq_886	*rgy1*	132.4	9	8	9
Fd7-Streptag		-	-	*fdx7*	11	9	34	3
Epsilon subunit PFOR	Porε	O67251	aq_1192a	*forD1*	9.1	8	58	6
**PFOR activity band**								
Alpha subunit PFOR	Porα	O67254	aq_1195	*forA1*	45.1	1130	73	27
Alpha subunit OGOR	Forα	O67229	aq_1167	*forA2*	42.7	722	70	28
Beta subunit PFOR	Porβ	O67255	aq_1196	*forB1*	32.2	459	59	16
Gamma subunit OGOR	Forγ	O67231	aq_1169	*forG2*	25.5	380	89	18
Delta subunit OGOR	Forδ	O67228	aq_1166	*aq_1166*	27.6	317	73	19
Beta subunit OGOR	Forβ	O67230	aq_1168	*forB2*	32.6	283	60	21
Gamma subunit PFOR	Porγ	O67256	aq_1200	*forG1*	26.7	260	61	16
Delta subunit PFOR	Porδ	O67252	aq_1192	*aq_1192*	21.3	249	65	11
Glutamate synthase small subunit		O67845	aq_2064	*gltD*	52.9	115	64	26
Epsilon subunit OGOR	Forε	O67232	aq_1171	*forD2*	9	75	73	8
Epsilon subunit PFOR	Porε	O67251	aq_1192a	*forD1*	9.1	30	58	6
Uncharacterized protein		O66853	aq_591	*aq_591*	39.2	29	45	14
Uncharacterized protein		O67227	aq_1163	*aq_1163*	14.9	19	65	8
Reverse gyrase 2		O67226	aq_1159	*rgy2*	134.5	16	13	14
Fd7-Streptag		-	-	*fdx7*	11	12	34	4

Table S3: Tandem mass spectrometry identification of proteins occurring in the bands revealed by PFOR and OGOR activity on the BN gel (Figure 4B, marked with a *). The 16 and 14 best hits are shown for the OGOR (in green) and the PFOR (in red) respectively, and the Fd7-Strep (20th best hit) was also reported for the PFOR. 38 and 72 proteins were identified in total in both bands. Accession: accession number in UniProt database. MW: theoretical molecular weight of the identified protein in kDa.

PSM: peptide spectrum match number (corresponding to the total number of identified peptide sequences for the protein, including those redundantly identified. Protein abundance approximation). COV: percent protein sequence coverage by the matching peptides. PEP: number of distinct peptides matching to protein sequence and unique to this protein.
